# STING-Expressing Neutrophils Facilitate Resolution of Inflammation After Myocardial Ischemia Reperfusion

**DOI:** 10.1016/j.jacbts.2026.101659

**Published:** 2026-10-26

**Authors:** Maegan L. Brockman, Triniti A. Scruggs, Jamarius P. Waller, Matthew A. Ensign, Julianna L. McNeice, Lanfang Wang, Rita Swinger, Samuel Dembowitz, Ivan Kordic, Raymond Dai, Rohini Polavarapu, Kaira R. Thevenin, Gabriella Kabboul, Rachel L. Kinsella, Ahmet F. Coskun, John W. Calvert, Rebecca D. Levit

**Affiliations:** a Division of Cardiology, Department of Medicine, Emory University School of Medicine, Atlanta, Georgia, USA; b Wallace H. Coulter Department of Biomedical Engineering, Georgia Institute of Technology and Emory University, Atlanta, Georgia, USA; c Department of Biochemistry, Emory University School of Medicine, Atlanta, Georgia, USA; d Parker H. Petit Institute for Bioengineering and Bioscience, Atlanta, Georgia, USA; e George W. Woodruff School of Mechanical Engineering, Georgia Institute of Technology, Atlanta, Georgia, USA; f School of Electrical and Computer Engineering, Georgia Institute of Technology, Atlanta, Georgia, USA; g Emory Vaccine Center, Emory National Primate Research Center, Emory University, Atlanta, Georgia, USA; h Division of Infectious Diseases, Department of Medicine, Emory University School of Medicine, Atlanta, Georgia, USA; i Interdisciplinary Bioengineering Graduate Program, Georgia Institute of Technology, Atlanta, Georgia, USA

**Keywords:** inflammation, neutrophils, reperfusion injury

## Abstract

Neutrophils are the earliest and most abundant immune cells to infiltrate the myocardium after myocardial ischemia/reperfusion (MI/R) injury. To date, the literature presents contradictory findings regarding the detrimental versus beneficial roles of neutrophils in MI/R. Activation of STING (Stimulator of Interferon Genes) signaling drives the expression of proinflammatory cytokines and type I interferons in immune cells. Here, we sought to investigate the role of neutrophil-intrinsic STING signaling during MI/R. Neutrophil-specific deletion of Sting resulted in significantly impaired cardiac function after MI/R, accompanied by reduced activation of NF-κB pathways in cardiac neutrophils, diminished early neutrophil recruitment to the myocardium, and defective neutrophil clearance. Immunostaining demonstrated decreased apoptosis in STING-deficient neutrophils, while flow cytometry showed reduced efferocytosis of these neutrophils by macrophages. Consistent with this, there was a reduction in anti-inflammatory macrophages within the myocardium 72 hours after MI/R. STING signaling in neutrophils is cardioprotective during MI/R by promoting efficient neutrophil clearance and facilitating the resolution of inflammation through the induction of anti-inflammatory macrophage polarization. Collectively, this work supports the concept that selectively targeting neutrophil effector function, rather than employing broad immunosuppression, represents a promising therapeutic strategy for MI/R.

## Highlights

• Neutrophil STING is critical for healing in MI/R.
• Macrophage efferocytosis of STING-deficient neutrophils is disrupted, altering post MI/R inflammation.
• Early innate immune responses shape cardiac outcomes, highlighting the need for targeted interventions.

## Visual Abstract

**Figure:**
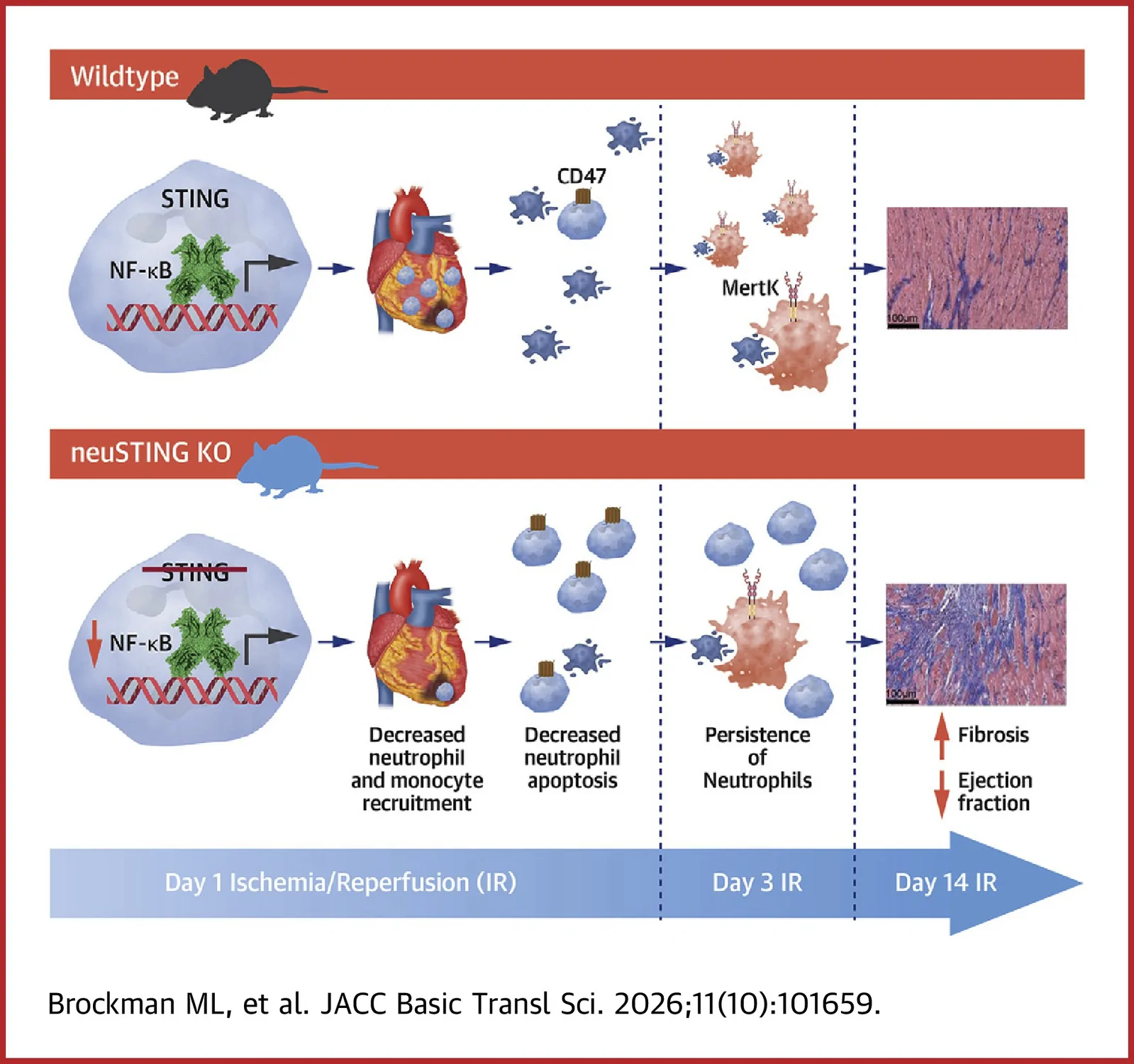

Ischemic heart disease is the leading cause of death worldwide, which often manifests as myocardial infarction, and is characterized by obstruction of coronary blood flow, leading to ischemia, tissue injury, and cardiomyocyte death.^1^^,^^2^ Paradoxically, after myocardial infarction, restoration of blood flow to the ischemic heart leads to damage in the form of myocardial ischemia/reperfusion (MI/R) injury. MI/R is inflicted through a multitude of mechanisms, including reactive oxygen mediated oxidative stress, activation of cell death pathways, and inflammatory immune responses. Collectively, these processes are thus an attractive target for improving cardiac function after MI/R.

Attempts to modulate inflammation after MI/R with immune-targeted therapies like colchicine and canakinumab have been shown to reduce cardiovascular events post-MI/R, but their benefits were tempered by an increased risk of infection without a clear improvement in survival.^3^, ^4^, ^5^, ^6^ Given the absence of effective treatments for immune-mediated MI/R, a deeper understanding of the underlying molecular and cellular targets is crucial for developing more effective interventions.

During MI/R, damage-associated molecular patterns (DAMPs) released from dying cardiomyocytes initiate a robust inflammatory cascade, of which the initiation is characterized by the rapid mobilization of neutrophils from the bone marrow and subsequent infiltration into the infarcted myocardium.^7^^,^^8^ Quality, quantity, location, and resolution of the MI/R immune response governs the quality of infarct and thus has a critical role in deciding post-MI/R cardiac function.^9^ Therefore, prolonged or dysregulated inflammatory responses after MI/R can result in excessive tissue injury, impaired healing, and adverse cardiac remodeling.^10^

The inflammatory phase is nonetheless an essential process after MI/R, as cells such as neutrophils are recruited to the infarct zone and clear the area of dead cells and matrix debris.^10^^,^^11^ Neutrophils can also cause further damage through the generation of reactive oxygen species (ROS), proteolytic enzymes, and neutrophil extracellular traps (NETs).^12^ Thus, neutrophils play a complex and dual role in MI/R by facilitating repair while also posing a risk for injury if overactive. Several studies have shown that attenuating neutrophil recruitment or activity can reduce infarct size and improve cardiac function.^13^, ^14^, ^15^ Conversely, multiple studies show that the initial neutrophil response influences many aspects of later inflammatory cell infiltration including cell type and number, and dampening the neutrophil response is detrimental to myocardial infarction healing.^16^^,^^17^ Accordingly, heterogeneity among neutrophil populations may help explain these seemingly contradictory findings in the heart, and emerging evidence suggests that early neutrophil responses can shape the recruitment, activation, and behavior of other immune cells, ultimately influencing the trajectory and resolution of inflammation.^8^^,^^16^

Stimulator of Interferon Genes (STING), a key regulator of type I interferon (IFN) transcription, is activated by the DNA sensor cyclic GMP-AMP synthase (cGAS), which detects cytosolic DNA and triggers STING signaling as a critical cellular defense mechanism against intracellular viral infection. Although STING is expressed at relatively low levels in neutrophils, its expression is sufficient to induce immune responses following pharmacological stimulation of STING.^18^ Furthermore, mice with constitutively active STING signaling in neutrophils had increased serum levels of proinflammatory cytokines.^18^ These data suggest that STING activation in neutrophils is sufficient to induce an inflammatory response mediated by cytokine release. Consistent with this, intratumoral STING activation has been shown to drive robust neutrophil accumulation through an NFκB/CXCR2-dependent mechanism, further underscoring the ability of STING signaling to shape neutrophil recruitment and function in vivo.^19^ In myocardial ischemia models, pharmacologic inhibition of STING with the small-molecule inhibitor H151 preserved myocardial function, reduced infarct size, and prevented excessive inflammation,^20^^,^^21^ and global inhibition of STING during myocardial infarction has been associated with dampened neutrophil priming in the bone marrow.^22^ However, these studies largely relied on global inhibitors or global knockout models, thus, characterization of neutrophil specific roles STING is still limited.

Therefore, we sought to investigate the role that neutrophil-specific STING plays in the development of MI/R injury. Here we show that neutrophil-specific STING knockout mice (hereby referred to as neuSTING KO) exhibited delayed resolution of the innate inflammatory response compared to wild-type (WT) mice and subsequently increased cardiac damage. Mechanistically, we found that STING-expressing neutrophils play a critical role in driving macrophage polarization toward reparative phenotypes, which is a key transition required for resolving inflammation following reperfusion, and minimization of damage after MI/R injury. These findings underscore the functional diversity of cardiac-infiltrating neutrophils and highlight the importance of neutrophil functions once traditionally viewed as harmful as critical events in healing.

## Methods

### Sex as a biological variable

For cardiac function studies, we examined male and female animals, and sex-dimorphic effects are reported. For all other studies, we exclusively examined male mice. It is unknown whether the findings are relevant for female mice.

### Animals

To assess the role of neutrophil-specific STING in MI/R, we generated a neutrophil-specific STING KO mice for use in experiments. We used the Catchup mouse strain (gifted from Dr Matthew Long) expressing Cre recombinase under the Ly6G promoter (Ly6G^Cre-tdTomato^), specific to murine neutrophils (Supplemental Figures 1A and 1B).^1^ These mice were crossed with mice having *loxP* sites flanking the *Sting* allele (STING^flox^, Jackson 031,670; C57BL/6J background) to generate the neutrophil-specific STING KO mice. Neutrophil-specific STING KO mice and aged-matched wildtype littermate controls (male or female; 10-12 weeks of age, genotype Ly6G^Cre−/+^, STING^flox/flox^ and Ly6G^Cre−/−^, STING^flox/flox^) were used. Animals were housed in the vivarium contained within Emory University's Division of Animal Resources with a 12-hour light-dark cycle and access to water and food ad libitum. All animal work was approved by the Emory University Institutional Animal Care and Use Committee (IACUC).

### Bulk sequencing of human neutrophils

All studies that involved drawing human blood samples were approved by the Emory University Institutional Review Board (STUDY00006169) and were done in accordance with institutional guidelines. All donors gave consent prior to blood draw. Ten milliliters of venous blood was collected through a 21-gauge needle in an EDTA containing Vacutainer (Becton, Dickinson, 366643). Neutrophils were isolated from whole blood using negative immunomagnetic selection (StemCell EasySep Direct Human Neutrophil Isolation Kit 19666). After isolation, 5 × 10^6^ cells were resuspended in Buffer RLT (Qiagen 79216) supplemented with β-mercaptoethanol. RNA was isolated (Qiagen RNeasy Mini 74104), and conventional bulk mRNA sequencing was performed using using Clontech SMARTseq v4, and sequencing on the NovaSeq6000 to a depth of 18 to 20 million reads/sample at 100SR in collaboration with the Emory Primate Center Genomics Core Laboratory. All donors were between the ages of 18 to 40; donor demographics are listed in Supplemental Table 10.

### Spatial transcriptomic analysis of human myocardial infarction samples

Spatial transcriptomic analysis of infarcted human heart samples was determined using deposited datasets from a recently published study.^22^ Patient characteristics are listed in Supplemental Table 11. Human myocardial tissue samples from infarcted (n = 2) and control (n = 2) hearts were analyzed using the 10x Genomics Visium spatial gene expression platform. Raw data were loaded using Seurat (v5.2.1), and each sample was assigned a unique identifier. Before integration, each sample underwent SCTransform normalization independently to account for technical variability. Normalized datasets were then merged and split by sample identity for batch-aware integration. Variable features were identified across samples, and a common set of 3,000 highly variable genes shared across datasets was selected for anchor-based integration using the SCTransform pipeline. Integration anchors were computed using the first 30 principal components. Dimensionality reduction was performed using principal component analysis (PCA) followed by uniform manifold approximation and projection (UMAP) for visualization. Clustering was conducted using a shared nearest neighbor (SNN) graph, and the Louvain algorithm with a resolution of 0.8. To annotate cell types, marker genes were identified and clusters were labeled based on canonical markers (Supplemental Table 1). For focused analysis of neutrophils, the neutrophil cluster was subset from the integrated object and reclustered at higher resolution, 1.0. Subclustering was visualized using UMAP, and marker genes for each neutrophil subcluster were identified.

Unless otherwise stated, data are presented as the mean ± standard deviation. Comparisons between groups were performed using Student's unpaired *t*-test, while comparisons among >2 groups used 1-way or 2-way analysis of variance (ANOVA) with Tukey's post hoc test for multiple pairwise comparisons. Repeated-measures 2-way ANOVA with Tukey's post hoc test was used to evaluate longitudinal changes. The assumption of normality was assessed using the Shapiro-Wilk test. A nonsignificant result (*P* > 0.05) indicated that the data did not significantly deviate from a normal distribution. If data were not normally distributed, the Wilcoxon rank-sum test was used for comparison. Statistical analysis was performed using GraphPad Prism 10.0.0 (GraphPad Software), and a *P* value of <0.05 was considered statistically significant.

Data and study materials supporting the findings of this study are available from the corresponding authors upon reasonable request. A detailed description of materials and methods can be found in the Supplemental Methods.

## Results

### STING is activated in human and murine neutrophils after MI/R injury

We first analyzed bulk RNA sequencing data from human neutrophils collected from 20 healthy male and 20 healthy female donors to confirm the expression of genes involved in STING activation (Figure 1A). Neutrophils isolated from male and female donors expressed transcripts for genes involved in STING activation through both the type I IFN and NFκB pathways (*STING, TBK1, IRF3, RELA, RELB*), confirming that human neutrophils express mRNA transcripts for components of the STING pathway.

**Figure 1:**
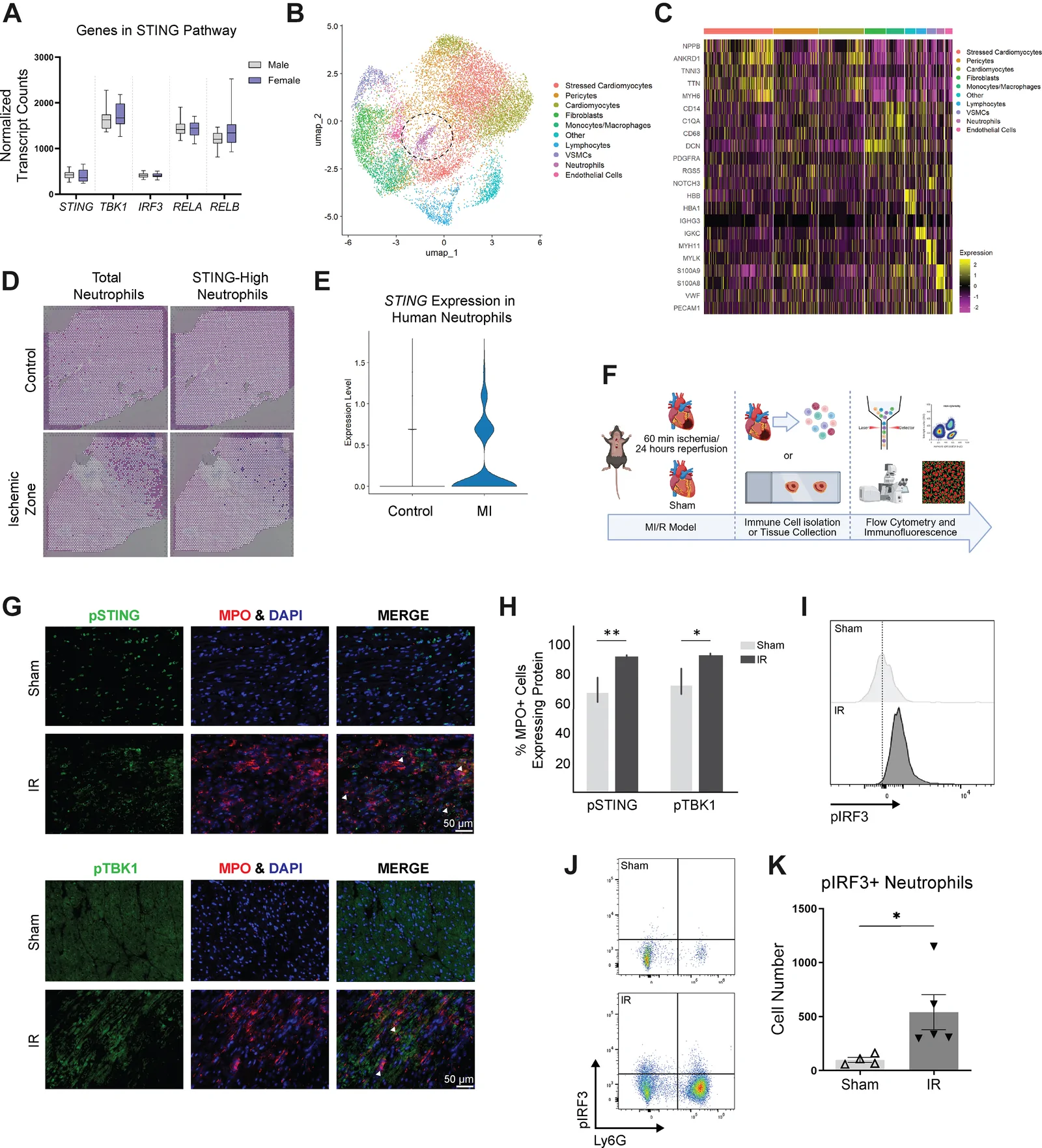
STING Is Activated in Human and Murine Neutrophils After Myocardial Ischemia/Reperfusion Injury (A) Bulk RNA sequencing of human neutrophils from healthy male and female donors showing genes expressed in the STING pathway. (B) UMAP plots derived from spatial transcriptomic analysis of samples taken from infarcted left ventricles after acute MI (n = 2) and control patients (n = 2).^23^ Neutrophils are indicated by the dashed circle. (C) Heat map plotting the top marker genes for each cluster. (D) Representative spatial plots from a control heart or the ischemic zone of an infarcted human heart showing total neutrophils (magenta color) and neutrophils which express high levels of *STING* transcripts (dark blue color). (E) mRNA expression of *STING* in neutrophils from control samples compared to neutrophils from MI samples. (F) Experimental scheme of MI/R in C57BL/6 mice. (G to H) Representative staining and quantification of pSTING (Ser366) and pTBK1 (Ser172) in neutrophils in the hearts of WT or sham mice or after 24 hours of reperfusion; (n = 3 per group). (I to K) Representative geometric mean fluorescence intensity (MFI), dot plot, and quantification of flow cytometry analysis of STING activation in neutrophils, as measured by IRF3 phosphorylation at S396, isolated from cardiac tissue 24 hours after MI/R compared to sham mice; (n = 4-5 per group). Data are presented as mean ± SD H, K. Statistical analysis was conducted using an unpaired t test H, K. ∗p<0.05; ∗∗p<0.01; ∗∗∗p<0.001; ∗∗∗∗p<0.0001.

Next, we analyzed published spatial transcriptomic data of human heart samples taken from the ischemic area of the left ventricle 2 days after myocardial infarction and respective control samples.^23^ The spatial data were normalized and unsupervised clustering revealed 10 distinct clusters, and cell type identities were assigned based on expression of canonical markers (Figures 1B and 1C, Supplemental Table 1). Of the 10 clusters, a neutrophil cluster was identified, and *STING* expression was evaluated in neutrophils. Neutrophils expressing high levels of *STING* were found to localize in the ischemic area after myocardial infarction (Figure 1D) and neutrophils from the ischemic, border, and remote zones were found to have higher expression of *STING* compared to neutrophils in control heart samples (Figure 1E).

Since gene expression does not always equate to pathway activation, we further sought to investigate STING activation in neutrophils in the setting of MI/R. Male C57BL/6J mice underwent either 60 minutes of ischemia followed by 24 hours of reperfusion or a sham thoracotomy, and cardiac tissue was subsequently collected for either immunohistochemistry or flow cytometric analysis (Figure 1F). We evaluated phosphorylation of STING at the S366 residue and TANK-binding kinase 1 (TBK1) at the S172 residue in neutrophils in heart tissues collected after MI/R. We found a significant increase in the percentage of pSTING^+^ and pTBK1^+^ neutrophils in the hearts of mice 24 hours after MI/R compared to mice that underwent the sham thoracotomy (Figures 1G and 1H). Next we assessed phosphorylation of interferon regulatory factor 3 (IRF3) at the S396 residue in neutrophils as a read out of STING activity, as STING associates directly with IRF3 after activation.^24^, ^25^, ^26^ Here, we demonstrate that pIRF3^+^ neutrophils are significantly increased in MI/R hearts compared to sham controls both in terms of cell number (Figures 1J and 1K) and in expression of pIRF3 (Figure 1I). Thus, these data identify for the first time a neutrophil population with increased STING expression and activation in the post-MI/R heart.

### Loss of STING in neutrophils worsens cardiac injury after MI/R

Having confirmed that STING is activated in neutrophils after MI/R, we sought to investigate the neutrophil intrinsic role of STING by generating a neutrophil-specific STING KO (neuSTING KO) mouse model, thus enabling us to further investigate if abrogating this pathway specifically in neutrophils could alter MI/R injury (Figure 2A, Supplemental Figures 1A and 1B). Both male and female neuSTING KO mice showed no differences in complete blood counts or weight compared to wildtype floxed (Ly6G^Cre−/−^, STING^flox/flox^) littermate controls (herein referred to as WT) (Supplemental Figures 1C to 1E). To validate our knockout efficiency, immune cells were isolated from the peritoneum of WT or neuSTING KO mice and neutrophils were isolated through density gradient centrifugation. This yielded 2 major populations: a non-neutrophil population containing macrophages, monocytes, and lymphocytes, and a purified neutrophil population for downstream studies. The purity of the neutrophil population was confirmed by flow cytometry (Supplemental Figure 2).

**Figure 2:**
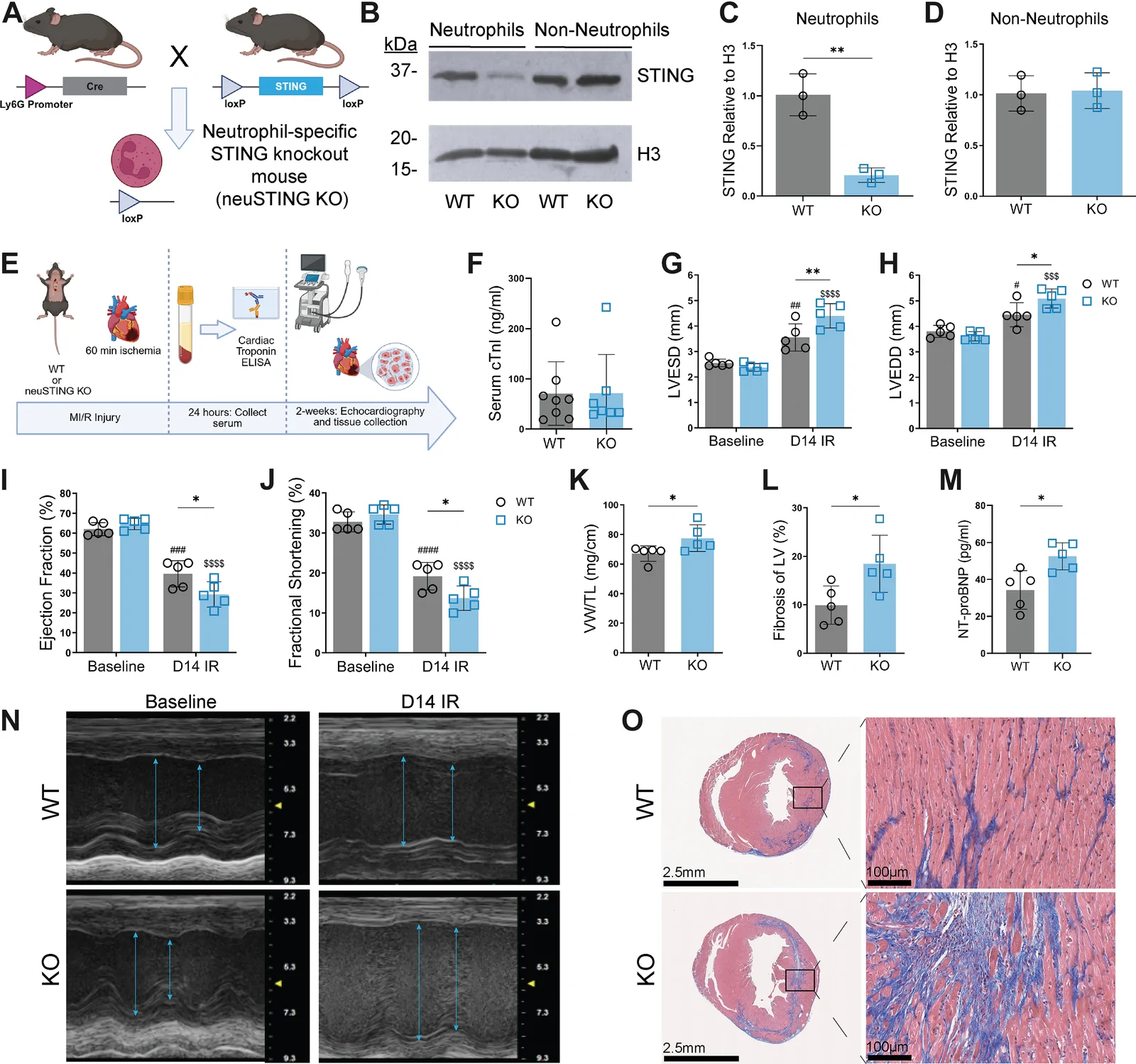
Loss of STING in Neutrophils Worsens Cardiac Injury After MI/R (A) Representative breeding strategy for the generation of neutrophil-specific STING knockout mice (neuSTING KO). (B to D) Representative immunoblot and quantification of STING knockout efficiency in neutrophils compared to non-neutrophils cells (monocytes, macrophages, and lymphocytes) isolated from the peritoneum (n = 3 per group). (E) Experimental scheme of cardiac function assessment. (F) Quantification of infarct size by serum troponin levels 24 hours after reperfusion in WT and neuSTING KO mice (n = 7-8 per group). (G to J) Echocardiographic assessment of left ventricular end-systolic diameter (LVESD), left ventricular end-diastolic diameter (LVEDD), ejection fraction, and fractional shortening in WT mice and neuSTING KO mice 14 days after reperfusion (n = 5 per group). (K) Ratios of ventricular weight to tibia length (VW/TL) in neuSTING KO mice compared to WT mice; (n = 5 per group). (L) Quantification of Masson's trichrome staining of the left ventricle in neuSTING KO mice compared to WT mice; (n = 5 per group). (M) Quantification of serum N-terminal pro–B-type natriuretic peptide (NT-proBNP) 14 days after reperfusion; (n = 5 per group). (N) Representative echocardiography images of parasternal long-axis left ventricular M-mode showing dilation of the left ventricle in neuSTING KO mice after MI/R injury. (O) Masson's trichrome staining of the left ventricle in neuSTING KO mice compared to WT mice (n = 5 per group). Scale bars = 2.5 mm and 100 μm. Data are presented as mean ± SD C to D and F to L. Statistical analyses were conducted using an unpaired *t*-test C to D, F and K to M and 2-way repeated-measures ANOVA with Tukey's post hoc test G to J. ∗*P* < 0.05, ∗∗*P* < 0.01, ∗∗∗*P* < 0.001, and ∗∗∗∗*P* < 0.0001; ^#^*P* < 0.05, ^##^*P* < 0.01, ^###^*P* < 0.001, and ^####^*P* < 0.0001 vs WT baseline; ^$^*P* < 0.05, ^$$^*P* < 0.01, ^$$$^*P* < 0.001, and ^$$$$^*P* < 0.0001 vs KO baseline. cTnI = cardiac troponin I; D14 = day 14; LV = left ventricle; MI/R = myocardial ischemia/reperfusion.

Western blotting analysis of neutrophils from neuSTING KO and WT mice confirms neutrophil-specific deletion of STING with an approximately 80% decrease in protein expression in neuSTING KO neutrophils only (Figures 2B to 2D). To further investigate the effects of neuSTING KO in cardiac remodeling, WT and neuSTING KO mice were subjected to MI/R injury (Figure 2E), and cardiac function was assessed 14 days after reperfusion. The assessment of serum cardiac troponin I (an indicator of infarct size^27^) at 24 hours of reperfusion revealed no significant difference between the groups (Figure 2F), suggesting that initial cardiomyocyte death was not affected by the deficiency of neutrophil STING.

Although 4 weeks post-MI/R is frequently used to evaluate cardiac function and remodeling, guidelines support 2 weeks as a valid and informative time point.^28^ We therefore selected this earlier time point to capture processes more closely aligned with the initial immune response to MI/R injury. In contrast to serum troponin levels, neuSTING KO mice showed significantly increased left ventricular end-systolic diameter and left ventricular end-diastolic diameter compared to WT mice 2 weeks post-MI/R (Figures 2G and 2H, Figure 2N), indicating greater dilatation of the left ventricle. Furthermore, neuSTING KO mice also showed significantly decreased ejection fraction and fractional shortening compared to WT mice (Figures 2I and 2J). Additionally, neuSTING KO mice also had increased ventricular weight to tibia length (VW/TL) ratios compared to WT mice, and Masson's trichrome staining for fibrosis revealed significantly increased fibrosis of the left ventricle in neuSTING KO mice (Figures 2K and 2L, Figure 2O). To further assess cardiac injury 14 days after reperfusion, we measured serum levels of N-terminal pro–B-type natriuretic peptide (NT-proBNP), a marker of cardiac stress and decompensation. Consistent with our echocardiographic findings, neuSTING KO mice had significantly increased serum levels of NT-proBNP compared to WT mice. Collectively, these data demonstrate that neuSTING KO mice have worse cardiac function following MI/R, and that the neutrophil intrinsic STING contributes to recovery of the heart after MI/R.

We also assessed the effects of loss of neutrophil-specific STING in MI/R injury in female mice (Supplemental Figure 3). No differences in cardiac function or fibrosis were observed between female WT and neuSTING KO mice (Supplemental Figures 3A to 3F) and correspondingly saw no difference in NT-proBNP levels between female WT and neuSTING KO mice (Supplemental Figure 3G). WT male and female mice showed no difference in the number of infiltrating neutrophils (Supplemental Figure 4A), but WT female mice had increased numbers of pIRF3^+^ neutrophils compared to WT male mice (Supplemental Figure 4B) 24 hours after MI/R. While simplistically this could imply female mice may have an exaggerated phenotype in response to STING activation, it is difficult to conclude if a compensatory pathway or other sex-based biological difference influenced the less dramatic phenotype seen in neuSTING KO female mice 2 weeks after MI/R. As such, male mice were chosen for subsequent experiments.

### Loss of STING in neutrophils increases cardiac type I interferon levels after MI/R

Because loss of STING in neutrophils resulted in worse cardiac function after MI/R, we wanted to assess if loss of STING in neutrophils affected the canonical type I IFN signaling pathway after MI/R. Thus, we assessed gene transcript levels through quantitative polymerase chain reaction of type I IFNs in cardiac tissue. Interestingly, type I IFN *Ifna1* and *Ifnb1* mRNA transcripts were increased in neuSTING KO mice compared to WT mice (Figures 3A and 3B); however, transcript levels of other inflammatory cytokines, such as *Tnf* and *Il6*, remained unchanged between groups (Figures 3C and 3D).

**Figure 3:**
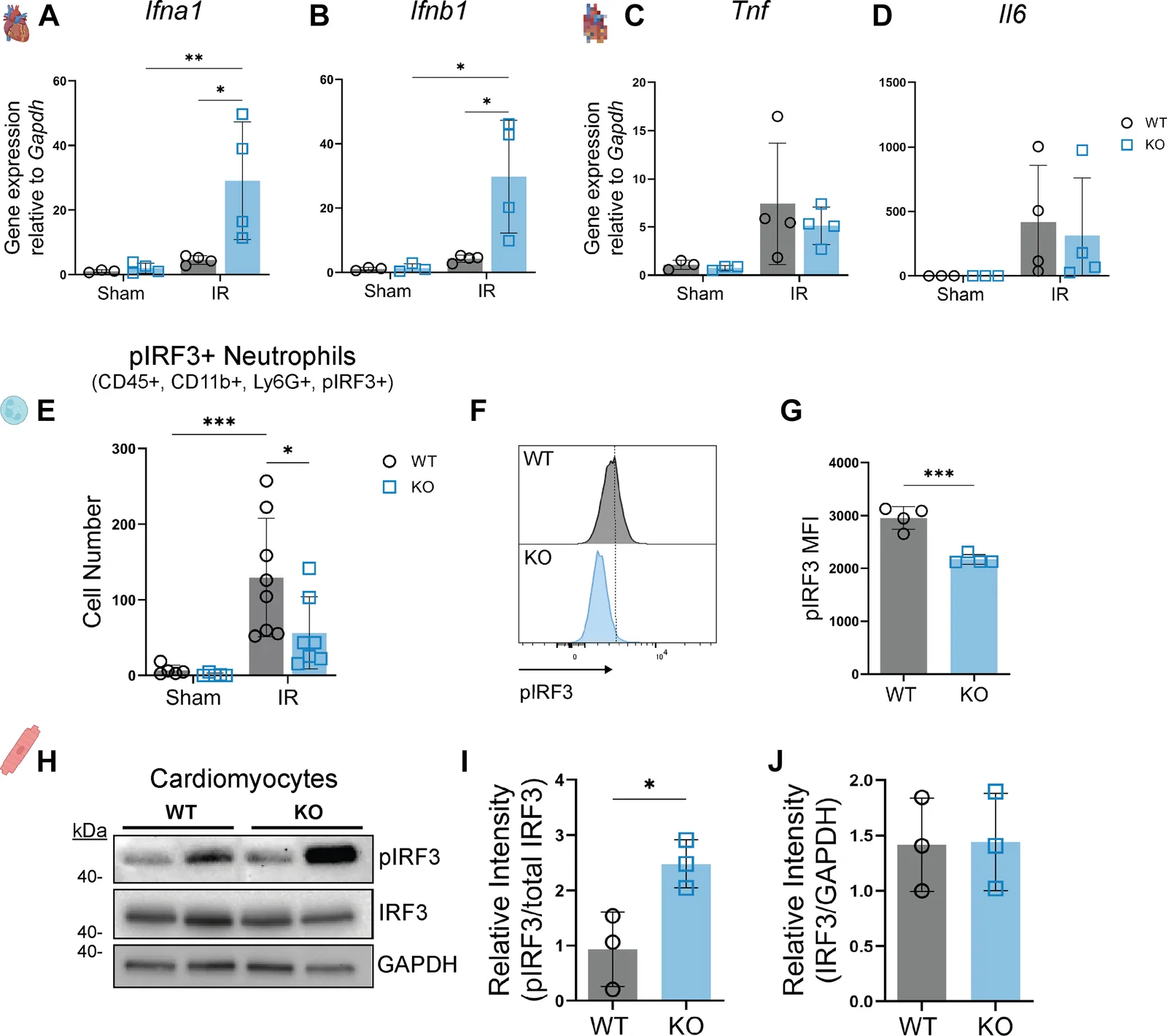
Loss of STING in Neutrophils Increases Cardiac Type I Interferon Levels After MI/R (A and B) Gene expression analysis of cardiac levels of *Ifna1* and *Ifnb1* in neuSTING KO mice compared to WT mice 24 hours after reperfusion (n = 3-4 per group). (C and D) Gene expression analysis of *Tnf* and *Il6* in neuSTING KO mice compared to WT mice 24 hours after reperfusion (n = 3-4 per group). (E) Flow cytometry analysis of cardiac pIRF3^+^ (S396) neutrophils in neuSTING KO mice compared to WT mice 24 hours after reperfusion (n = 5-8 per group). (F and G) The geometric mean fluorescence intensity (MFI) of pIRF3 in cardiac neutrophils from neuSTING KO mice compared to WT mice 24 hours after reperfusion (n = 4 per group). (H to J) Western blot analysis of pIRF3 (S396) levels in cardiomyocytes isolated 24 hours after reperfusion from WT and neuSTING KO mice (n = 3 per group). Data are presented as mean ± SD A to F and I to J. Statistical analyses were conducted using an unpaired *t*-test (F, I to J) and 2-way ANOVA with Tukey's post hoc test A to E. ∗*P* < 0.05, ∗∗*P* < 0.01, ∗∗∗*P* < 0.001, ∗∗∗∗*P* < 0.0001. MI/R = myocardial ischemia/reperfusion.

To assess STING response, we assessed pIRF3 levels in neutrophils by flow cytometry 24 hours post-MI/R and found that, as expected, neuSTING KO mice exhibited a significant reduction in both the percentage of pIRF3+ neutrophils (Figure 3E) and the geometric mean fluorescence intensity (MFI) of pIRF3 (Figures 3F and 3G). However, cardiomyocytes from neuSTING KO mice exhibited upregulation of pIRF3 (Figures 3H and 3J) 24 hours after MI/R, which is indicative of enhanced STING activation in cardiomyocytes in the neuSTING KO mice. These observations align with a recent study suggesting that type I IFNs are primarily produced by cardiomyocytes at the border zone after myocardial infarction, and suggests that the mechanism of neuSTING KO mice susceptibility to injury is not directly related to type I IFN signaling in neutrophils.^29^

### Single-cell RNA sequencing of neuSTING KO neutrophils reveals reduced inflammatory signaling after MI/R

To investigate potential pathways enriched in neuSTING KO neutrophils after MI/R, we performed single-cell RNA-sequencing analysis of noncardiomyocyte cell populations isolated from mice 24 hours after reperfusion (Figure 4A). The analysis included 22,467 cells from WT mice (n = 4 per group) with a median expression of 3,177 genes and 9,268 transcripts per cell, and 17,904 cells from neuSTING KO mice (n = 4 per group) with a median expression of 3,095 genes and 9,786 transcripts per cell. Visualization of these results through a UMAP plot revealed 15 distinct cell clusters (Figure 4B and Supplemental Figure 5, Supplemental Table 2). We then subset neutrophils from the integrated count matrices, and rescaled and reclustered the data, resulting in 4,177 WT neutrophils with a median expression of 1,400 genes and 4,682 transcripts per cell, and 3,779 neuSTING KO neutrophils with a median expression of 1,673 genes and 6,016 transcripts per cell (Figure 4C).

**Figure 4:**
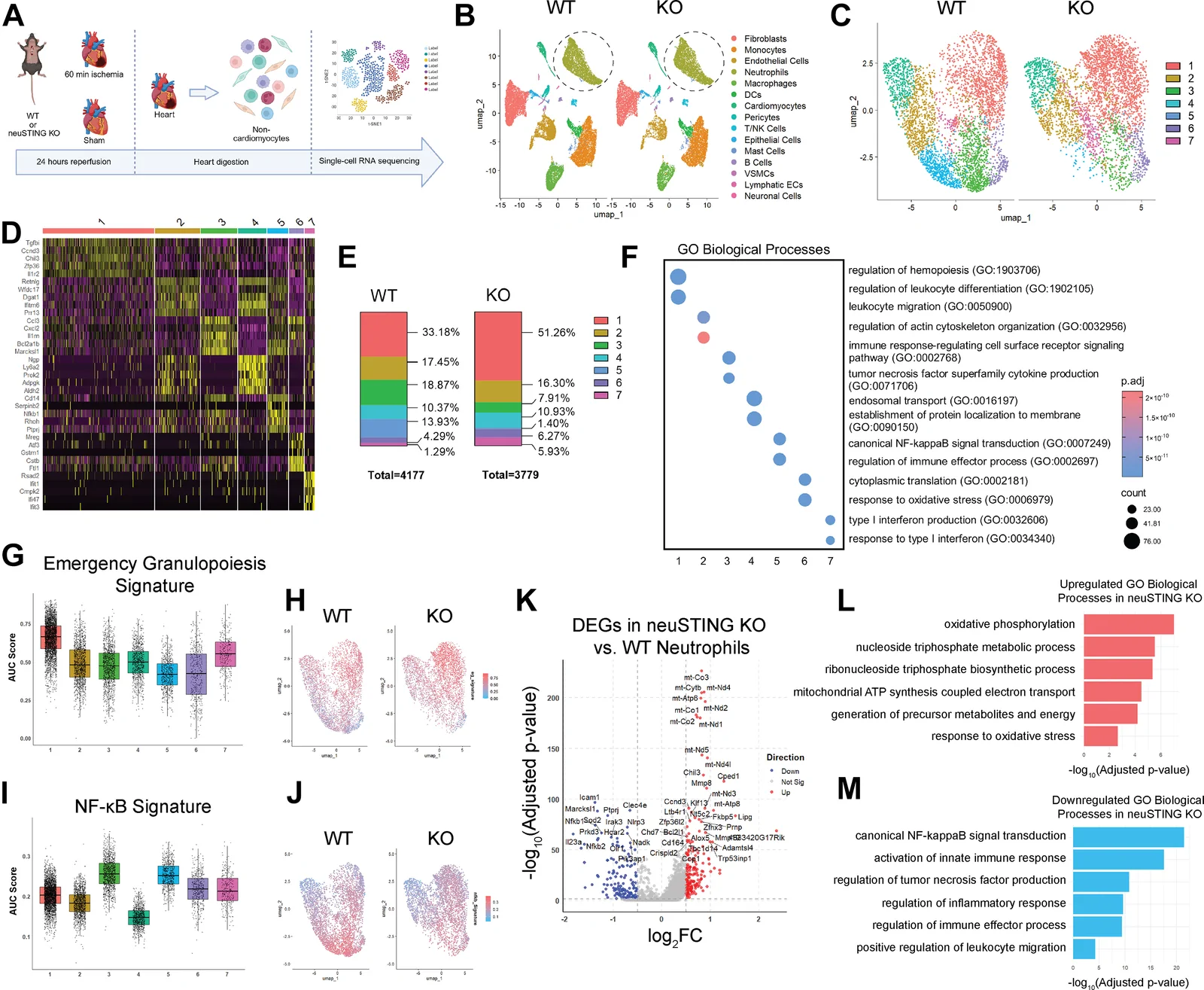
Single-Cell RNA Sequencing of neuSTING KO Neutrophils Reveals Reduced Inflammatory Signaling After MI/R (A) Experimental scheme of single-cell RNA sequencing experiment. (B) UMAP plots show 22,467 individual cells from WT mouse hearts (n = 4) and 17,094 individual cells from neuSTING KO mouse hearts (n = 4) with each cell type represented by a distinct color. Neutrophils are indicated by the dashed circle. (C) UMAP plots of cardiac neutrophils show 4,177 cells from WT hearts and 3,779 cells from neuSTING KO hearts, resolving into 7 distinct clusters. (D) Heat map of top marker genes across each neutrophil subcluster. (E) Proportions of neutrophil subclusters in cardiac neutrophils from WT and neuSTING KO mice. (F) Gene set enrichment analysis for GO (Gene Ontology) biological processes of top marker genes in neutrophil subclusters. The color of the dot indicates false discovery rate (FDR)–adjusted *P* value, and the size of the dot corresponds to the counts of genes in the indicated pathway. (G and H) NF-κB signature scores across neutrophil subclusters, with signature scores projected onto UMAPs of WT and neuSTING KO neutrophils. (I and J) Emergency granulopoiesis signature scores across neutrophil subclusters, with signature scores projected onto UMAPs of WT and neuSTING KO neutrophils. (K) Volcano plot of differentially expressed genes (DEGs) in cardiac neutrophils from neuSTING KO mice relative to WT mice. *P* values were corrected for multiple tests using the FDR method; criteria for differential expression were log_2_FC > 0.5 and adjusted *P* value < 0.05. Significantly upregulated genes are plotted in red, and significantly downregulated genes are plotted in blue. (L) Gene set enrichment analysis for GO biological processes identifying significantly enriched pathways in neuSTING KO cardiac neutrophils 24 hours after reperfusion compared to WT cardiac neutrophils. (M) Gene set enrichment analysis for GO biological processes identifying significantly downregulated pathways in neuSTING KO cardiac neutrophils 24 hours after reperfusion compared to WT cardiac neutrophils. AUC = area under the curve; MI/R = myocardial ischemia/reperfusion; UMAP = uniform manifold approximation and projection.

UMAP plots and a heat map of top marker genes identified 7 distinct neutrophil clusters (Figures 4C and 4D, Supplemental Table 3). The largest cluster, cluster 1, comprised 33% of WT neutrophils and more than half (51%) of neuSTING KO neutrophils (Figure 4E). This cluster was defined by genes enriched in regulation of hematopoiesis and leukocyte differentiation, suggesting that it represents emergency granulopoiesis in response to injury (Figure 4F). Cluster 2 contained 17% of WT and 16% of neuSTING KO neutrophils and was characterized by genes involved in migration and actin cytoskeleton organization, consistent with migratory capacity at the site of injury (Figures 4E and 4F). Clusters 3 and 5 were defined by genes involved in immune responses and NFκB signal transduction, representing inflammatory processes during MI/R (Figure 4F). Notably, both clusters were significantly reduced in neuSTING KO mice, with cluster 3 representing almost 19% of WT neutrophils and only 8% of neuSTING KO neutrophils, and cluster 5 representing 14% of WT neutrophils and only 1% of neuSTING KO neutrophils (Figure 4E). Cluster 6 was associated with translational machinery and made up 4% of WT and 6% of neuSTING KO neutrophils (Figures 4E and 4F). Lastly, cluster 7 was enriched for type I IFN signaling and response genes, a well-established neutrophil signature in myocardial ischemia^25^^,^^26^^,^^29^ (Figure 4F). Cluster 7 neutrophils accounted for 6% of neuSTING KO neutrophils, compared to 2% of WT, potentially reflecting neuSTING KO neutrophils responding to elevated type I IFN levels in the heart (Figure 3A).

The clusters that differed most in proportion between WT and neuSTING KO mice were clusters 1, 3, and 5. We therefore curated emergency granulopoiesis and NFκB signatures (Supplemental Table 7) and applied them across neutrophil clusters. Cluster 1 showed a strong emergency granulopoiesis signature, whereas clusters 3 and 5 showed strong NFκB signatures (Figures 4G to 4J). The near-absence of these proinflammatory NFκB clusters, clusters 3 and 5, in neuSTING KO mice thus may underlie an impaired or dysregulated immune response. Conversely, the expansion of cluster 1 in neuSTING KO mice may represent a compensatory mechanism in the absence of proinflammatory neutrophils.

Differential gene expression analysis revealed the most upregulated genes in neuSTING KO versus WT neutrophils (Figure 4K). Many downregulated genes corresponded to the reduced NFκB signature, whereas upregulated genes were largely mitochondrial (Figure 4K and Supplemental Table 4). Pathway analysis confirmed downregulation of canonical NFκB signaling and regulation of TNF production (Figure 4M and Supplemental Table 6), while upregulated pathways were enriched for oxidative phosphorylation (Figure 4L and Supplemental Table 5). Although neutrophils typically rely on glycolysis for energy, recent studies show that long-lived tumor-associated neutrophils can adapt metabolism to favor oxidative phosphorylation to persist in the tumor microenvironment.^30^^,^^31^ Hallmark analysis also revealed significant enrichment of STAT5 signaling genes in neuSTING KO neutrophils (Supplemental Table 5). Notably, STAT5 deletion in neutrophils has been linked to increased apoptosis,^32^ suggesting that STAT5 pathway activation may promote prolonged survival of neuSTING KO neutrophils after MI/R. Similarly, inhibition of NFκB signaling has been associated with enhanced neutrophil survival.^33^ Together, these data suggest that in the absence of STING, neutrophils exhibit muted inflammatory functions, an increased reliance on oxidative phosphorylation, and prolonged survival in the heart after MI/R.

### Loss of STING in neutrophils impairs cardiac neutrophil effector functions after MI/R

RNA sequencing revealed reduced NFκB signaling in neuSTING KO neutrophils 24 hours after MI/R, prompting us to assess common neutrophil effector functions in the absence of STING to determine the effects of altered inflammatory signaling in these cells. Because calcium is a central signaling mediator of neutrophil effector functions,^34^ we isolated bone marrow neutrophils from WT and neuSTING KO mice to assess calcium influx. Upon stimulation with W-peptide, which activates formyl peptide receptors and triggers calcium entry into the cytosol,^35^ neuSTING KO neutrophils exhibited a significantly reduced calcium influx compared to WT neutrophils (Figures 5A and 5B). Given this reduction, we hypothesized that additional neutrophil effector functions might also be impaired.

**Figure 5:**
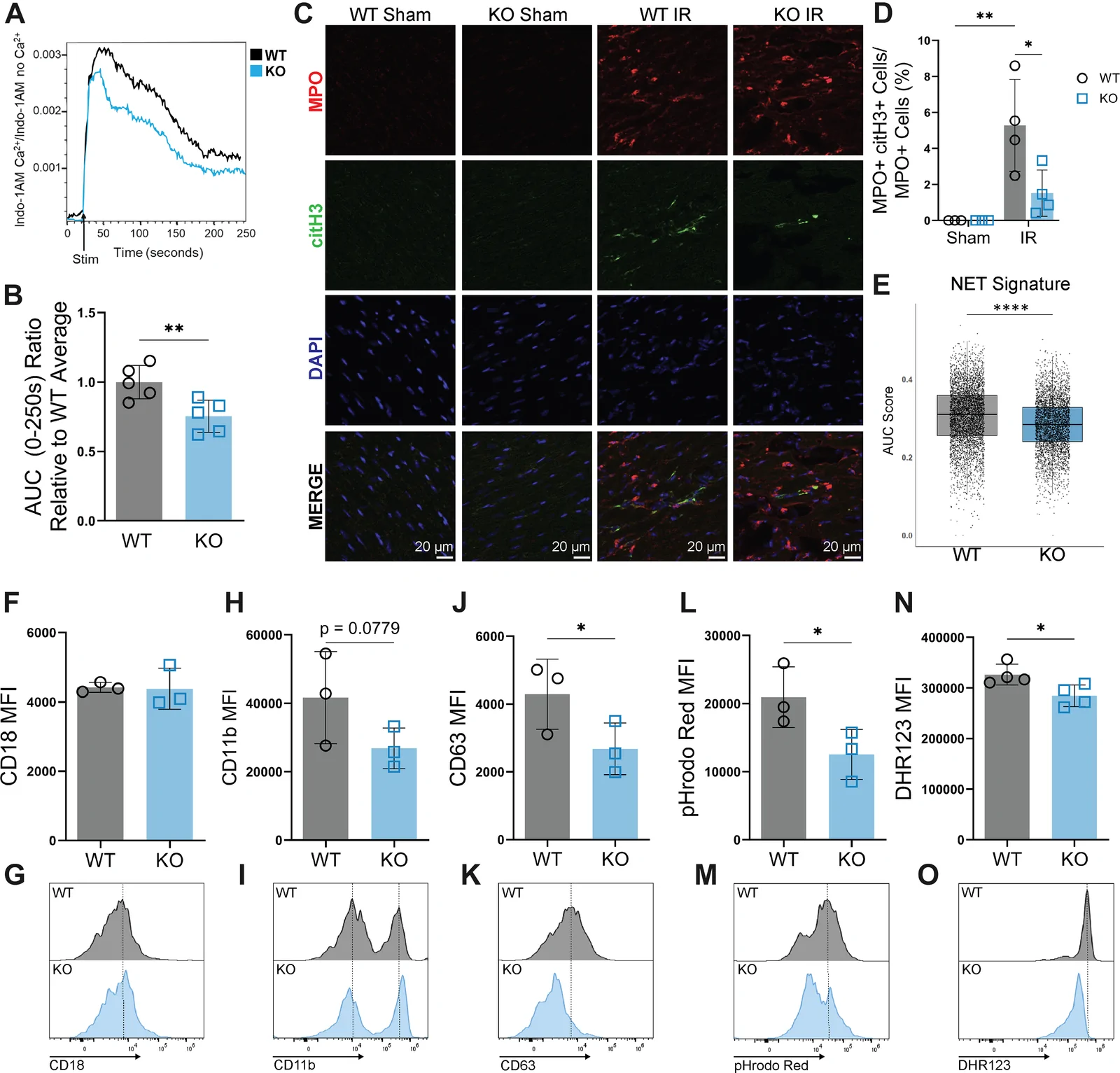
Loss of STING in Neutrophils Impairs Cardiac Neutrophil Effector Functions After MI/R (A and B) Representative trace of calcium flux in WT and neuSTING KO neutrophils after stimulation with W-peptide and quantification of the area under the curve (AUC) of calcium flux in WT and neuSTING KO neutrophils isolated from the bone marrow (n = 5 per group). (C and D) Representative staining and quantification of neutrophil extracellular traps (NETs) in WT and neuSTING KO hearts in sham mice or after 24 hours of reperfusion (n = 3-4 per group). (E) Comparison of NET signature scores in cardiac neutrophils from WT and neuSTING KO mice 24 hours after reperfusion. (F to K) Representative histograms plots and quantifications of neutrophil degranulation markers CD18, CD11b, and CD63 on cardiac neutrophils from WT and neuSTING KO mice 24 hours after reperfusion (n = 3 per group). (L and M) Representative histogram and quantification of phagocytosis of *Ecoli* particles in cardiac neutrophils from WT and neuSTING KO mice 24 hours after reperfusion (n = 3 per group). (N and O) Representative histogram and quantification of intracellular reactive oxygen species in cardiac neutrophils from WT and neuSTING KO mice 24 hours after reperfusion; (n = 4 per group). Data are presented as mean ± SD B, D, F, H, J, L, N; the median (center line), IQR (box), and 1.5 × IQR (whiskers); individual points represent single cells E. Statistical analyses were conducted using 2-way ANOVA Tukey's post hoc test D, unpaired *t*-test B, F, H, J, L, N, and Wilcoxon rank-sum test (E). ∗*P* < 0.05, ∗∗*P* < 0.01, ∗∗∗*P* < 0.001, ∗∗∗∗*P* < 0.0001. MI/R = myocardial ischemia/reperfusion.

We next assessed neutrophil extracellular trap (NET) formation through immunostaining of myeloperoxidase (MPO) and citrullinated H3 (citH3) in cardiac sections and found reduced citH3^+^ neutrophils in the hearts of neuSTING KO mice 24 hours after MI/R (Figures 5C and 5D). Similarly, we also saw a significantly reduced NET signature from our single-cell RNA sequencing data in neuSTING KO neutrophils compared to WT neutrophils (Figure 5E and Supplemental Figure 6B, Supplemental Table 7). We next assessed markers of neutrophil degranulation in cardiac neutrophils isolated from WT and neuSTING KO mice 24 hours after MI/R. CD18 and CD11b, which are upregulated with degranulation of secondary granules,^36^ were unchanged (Figures 5F to 5I). In contrast, CD63 deposition was reduced in neuSTING KO neutrophils (Figures 5J and 5K), indicating impaired primary granule release, which typically require stronger stimulation to promote degranulation. Degranulation signatures were not reduced in neuSTING KO neutrophils; however, neutrophil degranulation is difficult to assess solely based on gene expression (Supplemental Figure 6C).

We next evaluated phagocytosis and ROS production in cardiac neutrophils at 24 hours post-MI/R. NeuSTING KO neutrophils exhibited significantly reduced phagocytosis of *E* *coli* particles (Figures 5L and 5M) and lower intracellular ROS by DHR123 (Figures 5N and 5O), with ROS signatures localizing to clusters 3 and 5 (Supplemental Figure 6E). Together, these findings indicate diminished inflammatory effector functions in the absence of STING and underscore the importance of neutrophil activation for cardiac recovery after MI/R.

### Loss of STING in neutrophils dampens recruitment of neutrophils to the heart after MI/R

As loss of STING in neutrophils led to a reduction in neutrophil effector functions and resulted in a reduced inflammatory phenotype, we next examined how this altered inflammatory capacity influenced neutrophil dynamics following MI/R. We thus performed flow cytometry on immune cells isolated from the heart and blood of WT and neuSTING KO mice at 24 and 72 hours after MI/R (Figure 6A). All cells were gated on single and live cells, and the gating strategy is shown in Supplemental Figure 7. We found significantly fewer infiltrating leukocytes (CD45^+^) and myeloid cells (CD45^+^, CD11b^+^) in the myocardium of neuSTING KO mice compared to WT mice on day 1 after MI/R (Figures 6B and 6C).

**Figure 6:**
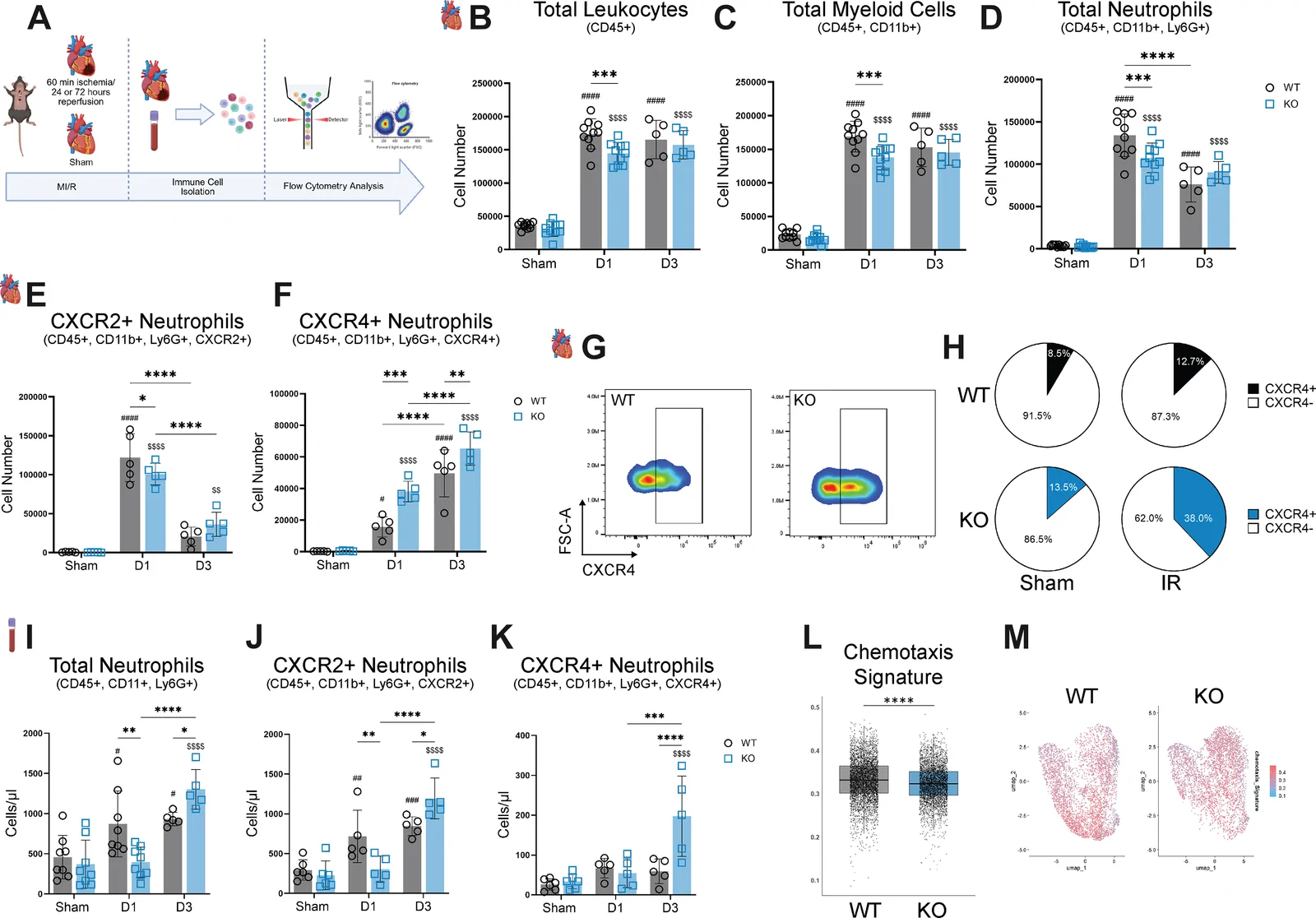
Loss of STING in Neutrophils Dampens Recruitment of Neutrophils to the Heart After MI/R (A) Schematic of experimental design showing timeline of MI/R and collection of immune cells for flow cytometry analysis, all following analysis was performed on single, live, cells. (B to D) Flow cytometry analysis of leukocytes (CD45^+^), myeloid cells (CD45^+^, CD11b^+^), and neutrophils (CD45^+^, CD11b^+^, Ly6G^+^) isolated from the myocardium of sham mice and mice at day 1 (D1) or day 3 (D3) after reperfusion in WT and neuSTING KO mice (n = 5-10 per group). (E and F) Flow cytometry analysis of CXCR2^+^ and CXCR4^+^ neutrophils isolated from the myocardium of sham mice and mice at D1 or D3 after reperfusion in WT and neuSTING KO mice (n = 5-6 per group). (G) Representative flow cytometry plot of CXCR4^+^ neutrophils in the hearts of WT and neuSTING KO mice at D1 after reperfusion. (H) Proportion of CXCR4^+^ neutrophils in the hearts of WT and neuSTING KO mice at D1 after reperfusion. (I to K) Flow cytometry analysis of total, CXCR2^+^, and CXCR4^+^ neutrophils in the blood of sham mice and mice at D1 or D3 after reperfusion in WT and neuSTING KO mice (n = 5-8 per group). (L) Comparison of chemotaxis signature scores in cardiac neutrophils from WT and neuSTING KO mice 24 hours after reperfusion. (M) Chemotaxis signature scores projected onto UMAPs of WT and neuSTING KO neutrophils. Data are presented as mean ± SD B to F and I to K; the median (center line), IQR (box), and 1.5× IQR (whiskers); individual points represent single cells L. Statistical analyses were conducted using 2-way ANOVA with Tukey's post hoc test B to F, I to K and Wilcoxon rank-sum test (L). ∗*P* < 0.05, ∗∗*P* < 0.01, ∗∗∗*P* < 0.001, and ∗∗∗∗*P* < 0.0001; ^#^*P* < 0.05, ^##^*P* < 0.01, ^###^*P* < 0.001, and ^####^*P* < 0.0001 vs WT sham; ^$^*P* < 0.05, ^$$^*P* < 0.01, ^$$$^*P* < 0.001, and ^$$$$^*P* < 0.0001 vs KO sham. MI/R = myocardial ischemia/reperfusion; UMAP = uniform manifold approximation and projection.

Likewise, there were significantly fewer neutrophils (CD45^+^, CD11b^+^, Ly6G^+^) in the hearts of neuSTING KO mice compared to WT mice on day 1 after MI/R (Figure 6D). After injury in WT hearts, the neutrophil population decreased in number from day 1 to day 3 post-MI/R; however, this decline was not observed in neuSTING KO hearts (Figure 6D), demonstrating persistence of neutrophils in heart in the absence of STING. We next looked at the markers CXCR2 and CXCR4 on cardiac and peripheral blood neutrophils. CXCR2 is a chemokine receptor essential for neutrophil egress from the bone marrow and migration to sites of injury, whereas CXCR4 has multiple roles, including promoting reverse migration to the bone marrow for clearance and mediating neutrophil retention in tissues.^37^ Further characterization revealed significantly fewer CXCR2^+^ neutrophils in neuSTING KO hearts at day 1 (Figure 6E), alongside a marked increase in CXCR4^+^ neutrophils at both day 1 and day 3 postreperfusion (Figures 6F to 6H).

Similar patterns were observed in peripheral blood, with neuSTING KO mice exhibiting significantly fewer total and CXCR2^+^ neutrophils in the blood at day 1, but by day 3, had significantly elevated levels of total, CXCR2^+^, and CXCR4^+^ neutrophils compared to WT (Figures 6I to 6K). Using the single-cell sequencing data of neutrophils from day 1 post-MI/R, we curated a chemotaxis signature based on genes involved in neutrophil chemotaxis (Supplemental Table 7) and applied it to WT and neuSTING KO neutrophils. In agreement with the flow cytometry data, neuSTING KO mice neutrophils displayed a significantly reduced chemotaxis signature (Figure 6L). Furthermore, feature plot analysis revealed high expression of chemotaxis genes in clusters 3 and 5, which are clusters that are decreased in neuSTING KO mice (Figure 6M). Collectively, these data show that loss of STING in neutrophils disrupts proper recruitment and clearance dynamics following MI/R, resulting in accumulation of neutrophils in the myocardium beyond the normal acute response window.

### Loss of STING in neutrophils decreases apoptosis and efferocytosis of cardiac neutrophils after MI/R

Given that neuSTING mice present with neutrophils persisting in the heart and blood on day 3, we hypothesized that neutrophils from neuSTING KO mice were not being cleared from the heart, and this resulted in inefficient resolution of inflammation. Efferocytosis of apoptotic neutrophils is necessary for the polarization of macrophages from pro- to anti-inflammatory and plays a critical role in resolution and healing following myocardial injury.^16^ Therefore, we set out to assess apoptosis and efferocytosis of neuSTING KO neutrophils.

We first evaluated apoptosis of neutrophils in the hearts of mice 24 hours after MI/R with immunofluorescence. MPO^+^ positive neutrophils were identified as apoptotic based on TUNEL positivity in tissue sections, whereas in flow cytometry, Ly6G^+^ neutrophils were assessed for apoptosis based on Annexin V staining. NeuSTING KO mice had significantly fewer TUNEL-positive neutrophils compared to WT mice, and neutrophils isolated from the myocardium 24 hours after MI/R showed reduced Annexin V staining in neuSTING KO mice compared to WT mice, confirming that loss of STING in neutrophils impairs apoptosis (Figures 7A and 7B, 7D and 7E).

**Figure 7:**
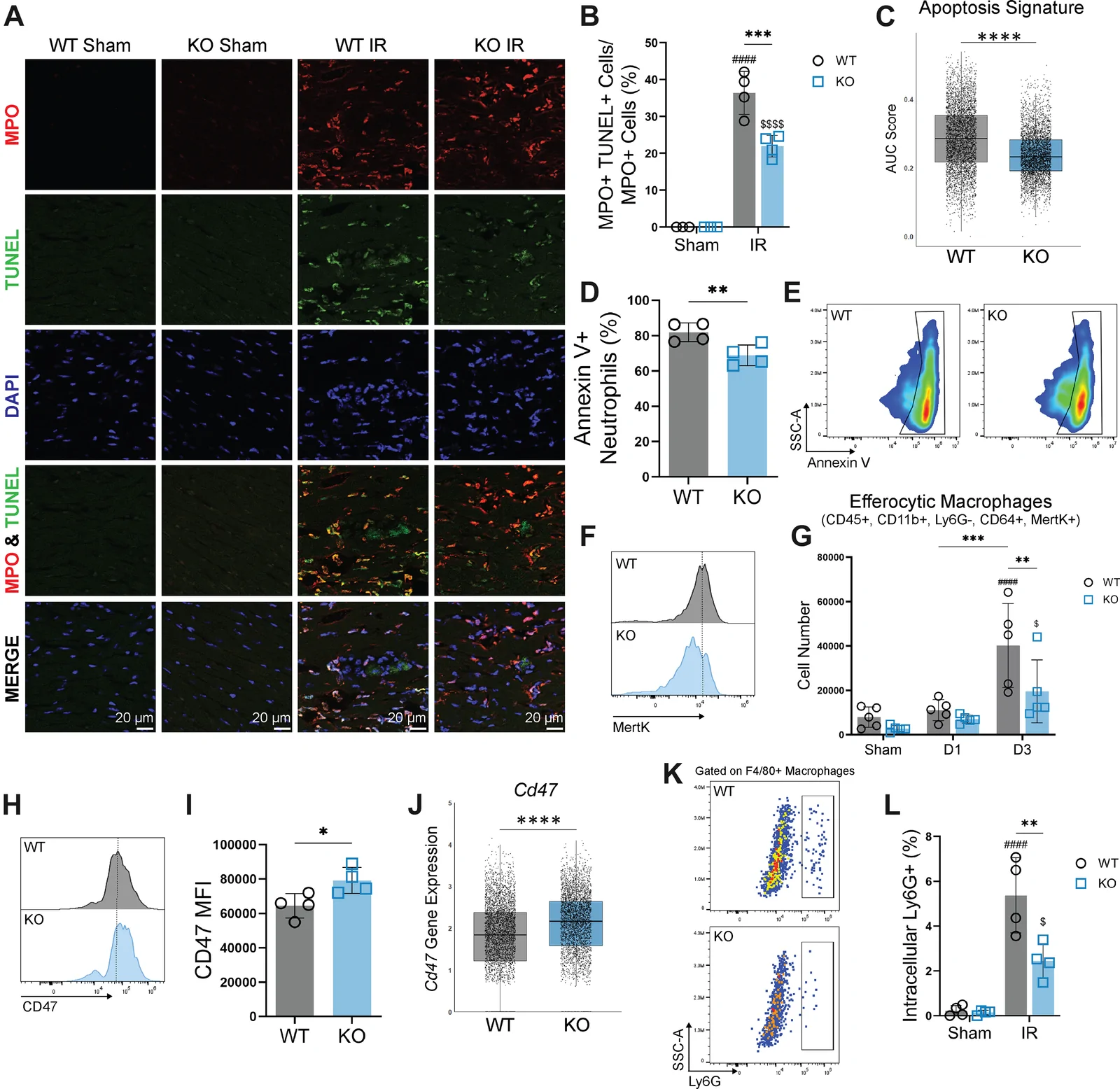
Loss of STING in Neutrophils Decreases Apoptosis and Efferocytosis of Cardiac Neutrophils After MI/R (A and B) Representative images and quantification of TUNEL staining in WT and neuSTING KO hearts in sham mice or after 24 hours MI/R (n = 3-4 per group). (C) Comparison of apoptosis signature score in cardiac neutrophils from WT and neuSTING KO mice 24 hours after reperfusion. (D and E) Quantification and representative flow cytometry plots of Annexin V staining in cardiac neutrophils from WT and neuSTING KO mice 24 hours after reperfusion (n = 4 per group). (F and G) Representative histogram and quantification of MertK^+^ macrophages isolated from the myocardium of sham mice and mice at day 1 (D1) or day 3 (D3) after reperfusion in WT and neuSTING KO mice (n = 5 per group). (H and I) Representative histogram and quantification of CD47 expression in cardiac neutrophils from WT and neuSTING KO mice 24 hours after reperfusion (n = 4 per group). (J) Comparison of *Cd47* expression in cardiac neutrophils from WT and neuSTING KO mice 24 hours after reperfusion. (K and L) Quantification and representative flow cytometry plots of intracellular Ly6G staining in F4/80^+^ macrophages in the hearts of sham mice or the hearts of mice 24 hours after reperfusion (n = 4 per group). Data are presented as mean ± SD B, D, G, I, K; the median (center line), IQR (box), and 1.5 × IQR (whiskers); individual points represent single cells (C, J). Statistical analyses were conducted using two-way ANOVA with Tukey's post hoc test B, G, K, unpaired *t*-test D, I, and Wilcoxon rank-sum test C, J. ∗*P* < 0.05, ∗∗*P* < 0.01, ∗∗∗*P* < 0.001, and ∗∗∗∗*P* < 0.0001; ^#^*P* < 0.05, ^##^*P* < 0.01, ^###^*P* < 0.001, and ^####^*P* < 0.0001 vs WT sham; ^$^*P* < 0.05, ^$$^*P* < 0.01, ^$$$^*P* < 0.001, and ^$$$$^*P* < 0.0001 vs KO sham. MI/R = myocardial ischemia/reperfusion.

Application of an apoptosis signature to neutrophils from single-cell sequencing data revealed a significantly lower score in neuSTING KO mice, with apoptosis genes again enriched in clusters 3 and 5 (Figure 7C, Supplemental Figure 8C, Supplemental Table 7). To further investigate impaired apoptosis, we also stained for CD47 on neutrophils, which functions as a “don't eat me” signal, preventing phagocytosis of CD47^+^ cells.^38^ Neutrophils isolated from the hearts of neuSTING KO mice 24 hours after MI/R expressed elevated levels of CD47, and single-cell sequencing confirmed increased *Cd47* gene expression in neuSTING KO neutrophils (Figures 7H to 7J). Assessment of CD47 at baseline in peripheral blood neutrophils shows no difference in the MFI of CD47 on neutrophils in WT and neuSTING KO mice before injury (Supplemental Figure 8A), suggesting that increased expression of CD47 on neuSTING KO neutrophils occurs in response to injury.

Given the decrease in apoptosis, we sought to investigate the efferocytotic capacity of macrophages in the heart at day 3 by assessing MER tyrosine kinase (MertK)^+^ positivity. We found that indeed, neuSTING KO mice had significantly decreased MertK^+^ macrophages in the heart at day 3 (Figure 7F and 7G). To confirm diminished efferocytosis of neuSTING KO neutrophils by macrophages in neuSTING KO hearts, we isolated immune cells from the hearts of mice 24 hours after MI/R and assessed the efferocytic capacity of macrophages. Macrophages were stained with F4/80 as an identity marker, and efferocytosis of neutrophils was assessed by intracellular staining for Ly6G, as macrophages that uptake neutrophils into their cytoplasm will stain positive intracellularly for neutrophil cell surface markers, including Ly6G.

Macrophages from neuSTING KO mice demonstrated significantly decreased intracellular Ly6G staining, indicating impaired efferocytosis (Figures 7K and 7L). Notably, there was a population of cells that were Ly6G+ and F4/80–, which we classified as neutrophils that were not engulfed by macrophages (Supplemental Figure 8B). To further confirm that loss of STING impaired neutrophil efferocytosis by macrophages, we performed an ex vivo efferocytosis assay, where bone marrow–derived macrophages were cocultured with apoptotic neutrophils from WT or neuSTING KO mice (Supplemental Figure 8D). Again, we demonstrated that neuSTING KO neutrophils were significantly less apoptotic, as measured by Annexin V staining (Supplemental Figure 8D). Furthermore, we showed that bone marrow–derived macrophages were unable to efficiently efferocytose neuSTING KO neutrophils (Supplemental Figure 8D). Taken together, these results show neutrophil-specific STING influences not only early neutrophil activity in the heart but also important downstream macrophage-driven events, specifically the performance of neutrophil efferocytosis by macrophages, a process critical for resolution of inflammation and cardiac recovery after MI/R.

### Loss of STING in neutrophils blunts monocyte recruitment and polarization of macrophages to an anti-inflammatory phenotype after MI/R

Loss of STING in neutrophils resulted in accumulation of cardiac neutrophils, reduced neutrophil apoptosis, and was accompanied by significantly fewer MertK+ macrophages after MI/R. These findings suggest defective efferocytosis; therefore, we hypothesized that STING deficiency limits efferocytosis of neutrophils and prevents macrophage polarization towards an anti-inflammatory phenotype. We thus assessed the dynamics of monocytes and macrophages after MI/R using flow cytometry. The number of total CD64^+^ cells in the heart at day 3 of reperfusion was significantly decreased in neuSTING KO mice compared to WT mice (Figure 8A). Interestingly, there was no significant difference in the number of proinflammatory macrophages (CD45^+^, CD11b^+^, Ly6G^−^, CD64^+^, CD206^−^) in the hearts of WT and neuSTING KO mice. However, there was a significant reduction in the number of anti-inflammatory macrophages (CD45^+^, CD11b^+^, Ly6G^−^, CD64^+^, CD206^+^) in neuSTING KO mouse hearts compared to WT at day 3 (Figures 8B and 8C). Proportionally, we observed a significantly greater percentage of proinflammatory macrophages and a lower percentage of anti-inflammatory macrophages in neuSTING KO mice at day 3 (Figures 8D and 8E). However, we did not see a difference in the ratio of anti- to proinflammatory macrophages at day 1 after reperfusion, suggesting the impairment of macrophage polarization occurs between days 1 and 3.

**Figure 8:**
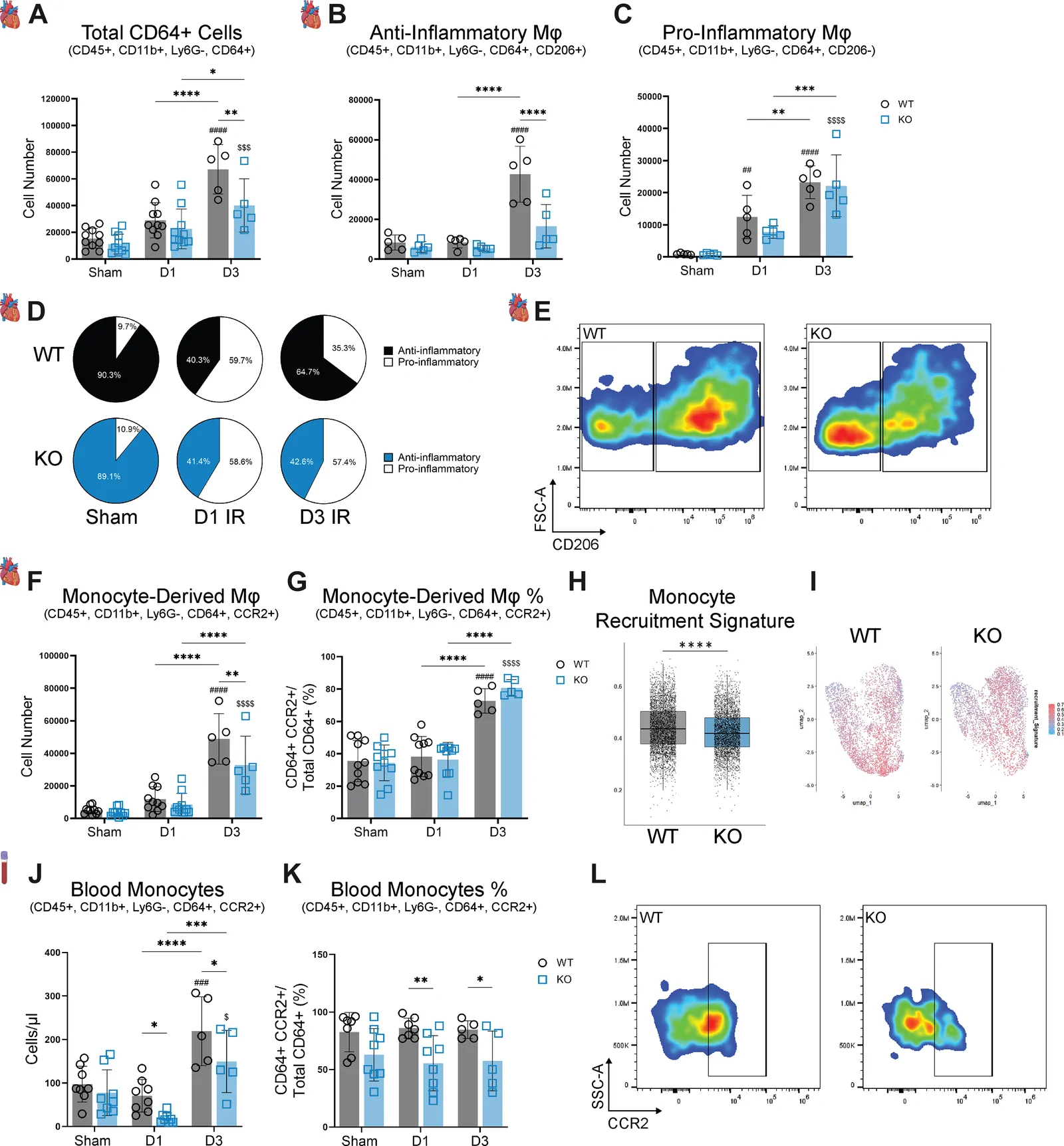
Loss of STING in Neutrophils Blunts Monocyte Recruitment and Polarization of Macrophages to an Anti-Inflammatory Phenotype After MI/R (A) Flow cytometry analysis of total CD64+ cells isolated from the myocardium of sham mice and mice at day 1 (D1) or day 3 (D3) after reperfusion in WT and neuSTING KO mice (n = 5-10 per group). (B and C) Flow cytometry analysis of anti- or proinflammatory macrophages isolated from the myocardium of sham mice and mice at D1 or D3 after reperfusion in WT and neuSTING KO mice (n = 5 per group). (D) Proportions of anti-inflammatory (CD206^+^) or proinflammatory (CD206^–^) macrophages in the hearts of WT and neuSTING KO mice at D1 and D3 after reperfusion. (E) Representative flow cytometry plot of CD206 expression on CD64^+^ cells in the hearts of WT and neuSTING KO mice at D3 after reperfusion. (F and G) Flow cytometry analysis of monocyte-derived macrophages isolated from the myocardium of sham mice and mice at D1 or D3 after reperfusion in WT and neuSTING KO mice (n = 5-10 per group). (H) Comparison of monocyte recruitment signature scores in cardiac neutrophils from WT and neuSTING KO mice 24 hours after reperfusion. (I) Monocyte recruitment signature scores projected onto UMAPs of WT and neuSTING KO neutrophils. (J and K) Flow cytometry analysis of monocytes in the blood of sham mice and mice at D1 or D3 after reperfusion in WT and neuSTING KO mice (n = 5-8 per group). (L) Representative flow plot of CCR2^+^ monocytes in WT and neuSTING KO mice at D1 after reperfusion. Data are presented as mean ± SD A to C, F to G, J to K; the median (center line), IQR (box), and 1.5 × IQR (whiskers); individual points represent single cells (H). Statistical analyses were conducted using 2-way ANOVA with Tukey's post hoc test A to C, F to G, J to K and Wilcoxon rank-sum test (H). ∗*P* < 0.05, ∗∗*P* < 0.01, ∗∗∗*P* < 0.001, and ∗∗∗∗*P* < 0.0001; ^#^*P* < 0.05, ^##^*P* < 0.01, ^###^*P* < 0.001, and ^####^*P* < 0.0001 vs WT sham; ^$^*P* < 0.05, ^$$^*P* < 0.01, ^$$$^*P* < 0.001, and ^$$$$^*P* < 0.0001 vs KO sham. MI/R = myocardial ischemia/reperfusion; UMAP = uniform manifold approximation and projection.

Flow cytometry studies revealed reduced numbers of proreparative macrophages by day 3 after M/IR, therefore, we analyzed the macrophage population within our single-cell data set to assess transcriptional changes at day 1 that may account for the changes seen at day 3. Macrophages were subset from the integrated count matrices, rescaled, and reclustered, which resulted in 1,751 macrophages from WT mice with a median expression of 3,267 genes with 9,503 transcripts per cell, and 1,216 macrophages from neuSTING KO mice with a median expression of 3,537 genes and 10,838 transcripts per cell. UMAP plots and a heat map of top marker genes revealed 6 distinct macrophage clusters (Supplemental Figures 9A and 9C).

Cluster 1, the largest cluster, comprised 30% of WT macrophages and 25% of neuSTING KO macrophages and corresponded to resident macrophages, as indicated by expression of the marker genes *Lyve1*, *Folr2*, and *Fgfr1* (Supplemental Figures 9B and 9C). This cluster was further characterized by enrichment of genes involved in receptor-mediated endocytosis and regulation of angiogenesis (Supplemental Figure 9D). Cluster 2 represented 28% of WT macrophages and 23% of neuSTING KO macrophages and corresponded to monocyte-derived macrophages, with enrichment of genes involved in leukocyte migration and actin filament organization (Supplemental Figures 9B and 9D). Cluster 3 comprised 17% of WT macrophages and 22% of neuSTING KO macrophages and was enriched for genes associated with antigen presentation. Cluster 4 comprised 18% of WT macrophages and 16% of neuSTING KO macrophages and was enriched for genes indicative of immune activation and pattern recognition receptor signaling (Supplemental Figures 9B and 9D). Cluster 5 represented only 4% of WT macrophages but 11% of neuSTING KO macrophages and was enriched for genes involved in responses to oxidative stress and endoplasmic reticulum (ER) stress. The final cluster, cluster 6, made up a small proportion of both WT and neuSTING KO macrophages and represented proliferative cells (Supplemental Figures 9B and 9D).

Differential expression analysis revealed upregulation of several inflammatory genes in neuSTING KO macrophages compared with WT macrophages, including *S100a8*, *S100a9*, and *Fcgr4* (Supplemental Figure 9E). Overall, these data suggest that neuSTING KO macrophages exhibit a more proinflammatory transcriptional profile than WT macrophages, which may help explain the reduced number of anti-inflammatory macrophages observed at day 3 in neuSTING KO mice. Notably, neuSTING KO macrophages exhibited a marked expansion of the stress-associated cluster 5, which was enriched for oxidative and ER stress pathways. This enrichment may reflect increased cytokine production and activation of inflammatory signaling pathways, consistent with a more synthetic, proinflammatory macrophage phenotype. Accordingly, these early transcriptional changes at day 1 may further shape subsequent macrophage responses, potentially amplifying the differences observed at day 3, when macrophage activity peaks.

We further examined the myeloid compartment in these mice by assessing monocyte-derived macrophages by using the marker CCR2 (CD45^+^, CD11b^+^, Ly6G^−^, CD64^+^, CCR2^+^). NeuSTING KO hearts had a significantly decreased number of monocyte-derived macrophages on day 3 compared to WT hearts (Figure 8F). However, the proportion of monocyte-derived macrophages to total CD64^+^ cells was not changed between the 2 groups (Figure 8G). When a monocyte recruitment signature was applied to cardiac neutrophils from WT and neuSTING KO mice, we found that neuSTING KO neutrophils had a significantly reduced monocyte recruitment score (Supplemental Table 7), with the highest scores observed in clusters 3 and 5. (Figures 8H and 8I). Likewise, there were significantly fewer circulating monocytes at days 1 and 3 in neuSTING KO mice (Figures 8J to 8L). These results indicate that loss of STING in neutrophils exerts cascading effects, blunting subsequent monocyte recruitment, monocyte-derived macrophage infiltration, and conversion of macrophages toward an anti-inflammatory phenotype.

## Discussion

Neutrophils are increasingly recognized as a heterogeneous cell type with diverse phenotypes that play multifactorial roles in health and disease. Recent studies have highlighted the importance of STING signaling in promoting cardiovascular injury^6^^,^^20^^,^^21^^,^^39^; however, the role of STING signaling in neutrophils in MI/R injury has remained poorly characterized. Our study identifies a novel role for STING in regulating neutrophil function and fate after MI/R injury. Neutrophil-specific STING deficiency delays resolution of the innate inflammatory response and exacerbates cardiac injury after MI/R. Mechanistically, STING-expressing neutrophils promote macrophage polarization toward reparative phenotypes, highlighting how early neutrophil activation exerts cascading effects on downstream immune responses.

Here, we demonstrate that STING is expressed in human and murine neutrophils and that it is activated after MI/R. Subsequently, we show that neutrophil-specific loss of STING leads to worsened cardiac remodeling, characterized by left ventricular dilation, fibrosis, and impaired contractility. Importantly, this impaired cardiac recovery occurred independent from initial cardiomyocyte death, as serum troponin was similar between groups. Thus, STING signaling in neutrophils influences postinjury inflammatory resolution rather than acute ischemic damage.

Canonically, STING regulates the production of type I INFs via the activation of IRF3. The worsened cardiac remodeling observed in neuSTING KO mice was not dependent on neutrophil-derived type I IFNs downstream of STING, as we observed reduced activation of IRF3 in neuSTING KO neutrophils after MI/R injury. However, we paradoxically observed elevated cardiac type I IFN levels to accompany the worse cardiac remodeling. We believe that this paradoxical increase in type I IFN is likely because of our findings of enhanced cardiomyocyte IRF3 activation, which is consistent with prior reports implicating cardiomyocytes as a dominant source of type I IFNs in myocardial infarction.^29^ In this study, sheer stress in the border zone led to nuclear rupture, release of DNA, and activation of STING and subsequent type I IFN production in border zone cardiomyocytes. Thus, lack of resolution of inflammation in neuSTING KO mouse hearts could exacerbate cardiomyocyte stress, leading to enhanced release of type I IFNs in the environment.

Single-cell RNA sequencing further revealed that STING-deficient neutrophils had significantly reduced NFκB signaling, with marked loss of proinflammatory clusters and enrichment of an emergency granulopoiesis population. This loss of NFκB activity aligns with prior studies linking impaired NFκB signaling to neutrophil survival and suggests that STING initiates neutrophil inflammatory programming through NFκB. Concomitantly, neuSTING KO neutrophils showed transcriptional enrichment of oxidative phosphorylation pathways, a metabolic profile atypical for short-lived, glycolysis-dependent neutrophils. This phenotype resembles tumor-associated neutrophils, which employ oxidative phosphorylation to persist in nutrient-poor environments. Inhibition of NFκB likewise promotes neutrophil persistence. Together, these findings suggest that loss of STING shifts neutrophils toward a long-lived, metabolically adapted phenotype with muted inflammatory capacity.

Functionally, these transcriptomic changes were reflected in impaired effector functions. NeuSTING KO neutrophils exhibited reduced calcium influx, diminished ROS production, decreased phagocytosis, and defective degranulation of primary granules, while secondary granule release remained intact. Flow cytometry revealed impaired recruitment and clearance dynamics, with fewer CXCR2^+^ neutrophils in the heart early after reperfusion, increased CXCR4^+^ neutrophils at later stages, and overall persistence of neutrophils beyond the acute response. Single-cell chemotaxis signatures were reduced in neuSTING KO neutrophils, again localizing to the inflammatory NFκB clusters lost in these mice.

Neutrophil persistence was further explained by defects in apoptosis and efferocytosis. NeuSTING KO neutrophils showed reduced Annexin V and TUNEL staining, lower apoptosis signature scores, and increased expression of the “don't eat me” signal CD47. These changes correlated with decreased efferocytosis, evidenced by reduced intracellular Ly6G in macrophages and fewer MertK^+^ macrophages in neuSTING KO mice. Because efferocytosis of apoptotic neutrophils is required for macrophage polarization toward an anti-inflammatory phenotype, its impairment therefore contributed to a lower proportion of reparative macrophages in neuSTING KO hearts. Accordingly, we observed significantly fewer monocytes, reduced monocyte-derived macrophages, and diminished anti-inflammatory macrophages in KO mice after MI/R, indicating that loss of neutrophil STING exerts cascading effects on subsequent innate immune responses.

Previous literature is conflicted on the role of STING and type I IFN signaling in cardiac injury after myocardial infarction. Prior studies have shown that blockade of STING and type I IFN signaling mitigates cardiac injury in myocardial infarction, while others have demonstrated no difference in injury between WT and global STING KO mice after myocardial infarction.^26^ We believe our findings help explain this discrepancy, as the detrimental effects of loss of STING in neutrophils may offset the favorable effects of STING deletion in other cell types, such as cardiomyocytes and macrophages. These results further highlight the complex role of inflammation, demonstrating that a balanced inflammatory response is essential for proper cardiac healing.

In this regard, the targeting of type I INF signaling after myocardial infarction is complicated by studies that have identified sex-based differences in type I INF responses. In humans, neutrophils from female donors have enriched type I IFN signaling,^40^^,^^41^ and in our model, male neuSTING KO mice displayed increased degree of injury after MI/R injury, while female neuSTING KO mice showed no difference in injury compared to WT controls. Given that it is known that female mice have reduced susceptibility to MI/R injury compared to male counterparts,^42^ more work on untangling the sex-based differences in STING signaling in both humans and mice is warranted.

### Study limitations

Although our study offers valuable insights into immune responses in models of acute mediated MI/R injury, several limitations should be acknowledged. First, the MI/R model used in mice does not fully reflect the clinical complexity of human myocardial infarction. Most patients present with preexisting comorbidities, such as atherosclerosis, hyperlipidemia, and hypertension, which significantly influence both baseline inflammation and postinfarction immune responses. Furthermore, while our model focuses on transient left anterior descending artery occlusion, many patients experience multivessel disease with blockages in various locations, leading to heterogeneous patterns of injury and immune activation that are not fully recapitulated in our approach.

Finally, important interspecies differences in immune cell biology should be considered. In humans, neutrophils constitute the majority of circulating leukocytes, whereas lymphocytes predominate in mice. Although neutrophil recruitment to the heart after injury is conserved, murine neutrophils differ from human neutrophils in key functional and transcriptional properties. These differences may influence both the kinetics and quality of the inflammatory response, and therefore, translational extrapolations should be made with caution.

## Conclusions

Neutrophils have long been recognized as double-edged swords in the context of MI/R injury. While their early recruitment is essential for clearing necrotic debris and initiating tissue repair, prolonged or excessive neutrophil presence can exacerbate cardiac damage. Various studies have attempted to modulate neutrophil recruitment after myocardial infarction, yielding mixed outcomes. For example, CXCR2 blockade or S100A9 inhibition has been shown to reduce infarct size and improve cardiac function in experimental models, yet other reports caution that complete neutrophil depletion may impair healing and worsen outcomes.^16^^,^^17^^,^^43^^,^^44^ Our findings highlight this complexity: neutrophil STING orchestrates the balance between inflammatory activation and timely clearance. In its absence, neutrophils fail to activate NFκB inflammatory pathways and evade apoptosis and efferocytosis, leading to their persistence in the myocardium. This dysregulated neutrophil phenotype has cascading effects on macrophage polarization and prevents effective resolution, ultimately worsening cardiac remodeling. By identifying STING as a checkpoint that couples neutrophil effector function with cell clearance, our study highlights a previously unrecognized mechanism of immune regulation after MI/R and raises the possibility that selectively targeting neutrophil STING could modulate postischemic inflammation and subsequent repair.

## Funding Support and Author Disclosures

Research in this study was supported by the National Institutes of Health (NIH) under award numbers P51OD011132 (to Dr Kinsella), R01HL140223 and R01HL175069 (to Dr Levit), R01DK115213 (to Dr Calvert), and R01HL164806 (to Dr Calvert); the NIH Training Grants T32-GM008602 (to Dr Brockman), 5T32HL007745-30 (to Dr Waller) and 5T32GM148391-03 (to Ms Thevenin); the Department of Defense under award number HT94252310420 (to Dr Calvert); and the American Heart Association under award numbers 24PRE1200255 (to Dr Brockman) and 25PRE1374554 (to Dr Kabboul). Dr Coskun holds a career award at the Scientific Interface from the Burroughs Wellcome Fund and Bernie-Marcus Early-Career Professorship. Dr Coskun was supported by start-up funds from the Georgia Institute of Technology and Emory University. Research reported in this study was supported by the National Institutes of Health under award number R35GM151028. This work is partially supported by the National Science Foundation CAREER under grant number 2338935. The authors have reported that they have no relationships relevant to the contents of this paper to disclose.

Perspectives **COMPETENCY IN MEDICAL KNOWLEDGE:** Ischemic heart disease is the leading cause of cardiovascular death and there is currently no therapeutic strategy to mitigate acute mediated myocardial ischemia/reperfusion injury.

**TRANSLATIONAL OUTLOOK:** Neutrophil-intrinsic STING signaling promotes neutrophil clearance and anti-inflammatory macrophage polarization, contributing to improved cardiac outcomes after MI/R injury. These findings support cell-specific targeting of neutrophil STING activity as a therapeutic approach, rather than broad immunosuppression, to modulate postinjury inflammation.
