# Supplementary material for: STING-Expressing Neutrophils Facilitate Resolution of Inflammation After Myocardial Ischemia Reperfusion
Source: JACC Basic Transl Sci. 2026 Oct 26;11(10):101659. doi: 10.1016/j.jacbts.2026.101659 (PMC13749089; doi:10.1016/j.jacbts.2026.101659)
Supplement: Supplementary file 1 [file mmc1.docx]

**Supplemental Material**

**STING Expressing Neutrophils Facilitate Resolution of Inflammation after Myocardial Ischemia Reperfusion**

Maegan L. Brockman, PhD^1^, Triniti A. Scruggs, BS^1^, Jamarius P. Waller, MD, PhD^1^, Matthew A. Ensign, BS^1,5^, Julianna L. McNeice, BS^1^, Lanfang Wang, MS^1^, Rita Swinger, BS^2^, Samuel Dembowitz, MS^5,8^, Ivan Kordic, MS^5-8^, Raymond Dai, BS^2^, Rohini Polavarapu, BS^2^, Kaira R. Thevenin, BS^4^, Gabriella Kabboul, BS^1,5^, Rachel L. Kinsella, PhD^3,4^, Ahmet F. Coskun, PhD^5,8,9^, John W. Calvert, PhD^2*^, Rebecca D. Levit, MD^1*^

This file includes:

Supplemental Methods

Supplemental Tables S8-S11

Supplemental Figures S1-S9

*Supplemental Table S1-S7 are available as separate word files

**Methods:**

*Genotyping:*

Ear biopsies of mice 4-6 weeks old were incubated at 55 °C for overnight in DirectPCR lysis buffer (Viagen 402-E) containing 0.3 mg/ml proteinase K (Thermo AM2546). The enzyme was heat inactivated by incubating samples at 85° for one hour after digestion. Primers used for the detection of the wild-type Ly6G allele were: Forward 5′-GGTTTTATCTGTGCAGCCC-3′; Reverse 5′-GAGGTCCAAGAGACTTTCTGG-3′) and for the detection of the mutant Ly6G allele were: Forward 5′-ACGTCCAGACACAGCATAGG-3′; Reverse 5′-GAGGTCCAAGAGACTTTCTGG-3′). Primers used for the detection of the floxed STING allele were: Forward 5’-TTTTCATCTGCCTTCCAGGT-3’; Reverse 5’-GCGCACACACACTAAAAACTG-3’. Primers and DNA samples were added to a PCR Master Mix (Thermo K0171). The following PCR conditions were applied: 1 min, 95 °C initial denaturation; 15 sec, 95 °C cyclic denaturation; 15 sec, 60 °C cyclic annealing; 35 sec, 72 °C cyclic elongation for a total of 35 cycles, followed by a 10-min 72 °C elongation step. PCR amplification products were analyzed by agarose gel electrophoresis.

*Myocardial Ischemia Reperfusion Model:*

Mice 10-12 weeks of age were anesthetized with intraperitoneal injections of ketamine (80-100 mg/kg) and xylazine (5-10 mg/kg), intubated, and connected to a rodent ventilator. Prior to the surgical procedure, mice also received an intraperitoneal injection of 200U/kg sodium heparin. A median sternotomy was performed, and the proximal left coronary artery was ligated with a 7-0 silk suture for a duration of 60 minutes. A short segment of PE-10 tubing was placed between the LCA and 7-0 silk suture to minimize damage and allow for complete reperfusion following the ischemic period. After 60 minutes, the ligature and tubing were removed, and reperfusion was confirmed. The chest wall and skin incision were closed with 4-0 Monocryl suture and a monofilament suture respectively. Mice were given buprenorphine extended-release (0.5mg/kg) as an analgesic after surgery and 72 hours post-surgery if needed.

*Echocardiographic Analysis:*

Mice were anesthetized with isoflurane in 100% O_2_ (5% for induction and 1-1.5% for maintenance) and secured to the Visualsonics Rail System III with medical tape. Core body temperature, respiration rate, and EKG were continuously monitored throughout the procedure. Transthoracic echocardiography of the left ventricle using a scan head interfaced with a Vevo echocardiography system (Visualsonics) was used to obtain high resolution B-mode and M-mode images.

*Histology (Masson’s Trichrome Staining):*

Tissues were fixed overnight in 10% formalin, routinely processed, paraffin-embedded, and sectioned at 5µM. Paraffin embedded sections were subjected to deparaffinization in xylene, rehydrated in a graded series of ethanol, rinsed with deionized water, and fixed in Bouin's Solution (Sigma HT10132) at room temperature overnight. Slides were then rinsed in a container with running tap water until clear, then rinsed once in diH2O and stained with Weigert’s Iron Hematoxylin solution (Sigma 1159730002) for 5 minutes. Slides were rinsed in tap water and then diH2O and stained with Biebrich Scarlet-Acid Fuschin (Sigma HT151) for 5 minutes. Rinsing in tap water then diH2O was repeated, followed by 5 min in 5% phosphotungstic/phosphomolybdic acid solution for 5 min (Sigma HT152 and HT153 respectively). Slides were rinsed in diH2O then stained for 4 min in Aniline Blue solution, then rinsed again in diH2O, and set in 1% acetic acid for 2 min. Slides were then dehydrated by consecutive two dips in diH2O, 75%, 95%, and 100% ethanol, then twice in xylene, and mounted using Cytoseal (Richard-Allan Scientific 8310–4). Images were taken at 40x with a NanoZoomer SQ Whole Slide Scanner (Hamamatsu) and analyzed using ImageJ software.

*Serum Troponin and NT-proBNP ELISAS:*

Infarct size was quantified measured through the serum concentration of cardiac troponin (cTnI), as is used clinically. Blood was collected from the inferior vena cava, centrifuged at 3,000 x g for 10 minutes. Serum was assayed for cTnI using High Sensitivity Mouse Cardiac Troponin ELISA kit (Life Diagnostics CTNI-HSP) according to the manufacture’s protocol. For NT-proBNP measurements, serum was collected as described above 14 days after reperfusion. Serum was assayed for NT-proBNP using the Abcam kit ab324412 according to the manufacture’s protocol.

*Isolation of Cardiomyocytes and Non-cardiomyocytes:*

Adult mouse cardiomyocytes and non-cardiomyocytes were isolated using a combination of a Langerdorff-free and Langendorff method^2, 3^. Briefly, mice were anesthetized (2-5% isoflurane in room air) and the heart was exposed by opening the chest cavity. After cutting the descending aorta, the heart was flushed with EDTA buffer (130 mM NaCl, 5 mM KCl, 0.5 mM NaH2PO4, 10 mM HEPES, 10 mM glucose, 10 mM BDM, 10 mM taurine, 5 mM EDTA, pH 7.8) through the right ventricle. The heart was then removed, and a blunt 27-gauge needle was inserted through the aorta into the left ventricle. The aorta and needle were clamped and then secured on a Langendorff apparatus. Digestion of the heart was achieved by sequential perfusion of EDTA buffer, perfusion buffer (130 mM NaCl, 5 mM KCl, 0.5 mM NaH2PO4, 10 mM HEPES, 10 mM glucose, 10 mM BDM, 10 mM taurine, 1 mM MgCl2, pH7.8), and collagenase buffer (0.5 mg/mL collagenase 2, 0.5 mg/mL collagenase 4, and 0.05 mg/mL protease XIV in perfusion buffer) at 37 °C. The atria and right ventricle were removed and left ventricle was gently disaggregated by teasing apart the tissue into 1-mm pieces using forceps. Cellular dissociation was completed by gentle trituration, and enzyme activity was inhibited by addition of stop buffer (5% sterile FBS in perfusion buffer). The cell suspension was then passed through a 100-μm filter. The cardiomyocytes and nonmyocytes were separated by gravity settling. The cell pellet was enriched with myocytes, whereas the supernatant contained nonmyocyte cardiac populations.

*Gene Expression Analysis of Cardiac Interferons and Cytokines:*

Quantitative real-time PCR (qRT-PCR) was performed to measure the expression levels of the type I interferons and interferon stimulated genes in cardiac tissue. RNA was isolated using RNeasy Kits for RNA Purification (Qiagen 74106). Synthesis of cDNA was performed using High-Capacity cDNA Reverse Transcription Kit's (Thermo Fisher Scientific 4374966) protocol. The housekeeping gene was used as an endogenous control and all genes expressed were normalized to the *Gapdh* housekeeping gene using the ddCT method. The primers used are listed in Table 8.

*Western Blot:*

Neutrophils isolated from the peritoneum of WT and STING KO mice were collected and lysed in RIPA buffer supplemented with protease inhibitors. Cardiomyocytes were collected as described above and lysed in RIPA buffer supplemented with protease inhibitors. Protein concentrations were measured with the Pierce™ BCA Protein Assay Kits (Thermo Scientific 23209). Equal amounts of protein were loaded into lanes of 12% SDS-PAGE gels and subsequently transferred to a polyvinylidene difluoride membrane. The membranes were then blocked and probed with primary antibodies at 1:1000 dilution (STING (D1V5L): Cell Signaling 50494; H3 (1B1B2): Cell Signaling 14269; IRF3 (D83B9): Cell Signaling 4302; pIRF3 (Ser396; 4D4G): Cell Signaling 4947) overnight at 4°C. For GAPDH ((GA1R) Invitrogen MA5-15738-D680), membranes were probed with antibody at 1:5000 dilution for one hour at room temperature. Immunoblots were next processed with secondary antibodies for 1 hour at room temperature. For STING and H3, immunoblots were probed with ECL Western Blotting Substrate (Thermo Scientific PI32209) to visualize signal, followed by visualization with film. Data were analyzed using ImageJ. Protein levels of STING were normalized to the housekeeping genes H3. For pIRF3 and IRF3, immunoblots were probed with SuperSignal™ West Pico PLUS Substrate (Thermo Scientific 34578) to visualize signal, followed by visualization with ChemiDoc™ MP Imaging System (Bio-Rad). Data were analyzed using Image Lab 6.1 (Bio-Rad). Protein levels of pIRF3 and IRF3 were normalized to GAPDH and the normalized ratios were analyzed.

*Single-Cell RNA Sequencing (Sample Preparation, Library Preparation, Sequencing, Alignment):*

Cells were isolated from the heart following MI/R as described above. After isolation, cells were washed, resuspended in RPMI, and layered onto a 75%/40% Percoll gradient (Sigma, P1644) to remove debris. The gradient was centrifuged at 1,300 × *g* for 20 minutes at 4°C. Following centrifugation, the interphase between the 40% and 75% layers was collected and pelleted. Cells were then washed and resuspended in PBS supplemented with 2% fetal bovine serum (FBS) for library construction. Libraries were constructed by the Emory University Integrated Genomics Core (EIGC) using the 10x Genomics Chromium GEM-X platform and gene expression libraries were then sequenced using an Illumina NovaSeq X Plus targeting a depth of 50,000 reads per cell. Cell Ranger software (10x Genomics) was used for low-level analysis, including demultiplexing, mapping to a reference transcriptome, and annotation of unique molecular identifiers.

*Single-Cell RNA Sequencing (Quality Control, Dimension Reduction, Clustering)*

Raw sequencing data were processed using the 10x Genomics Cloud Analysis platform. Alignment, barcode processing, and UMI counting were performed with Cell Ranger (v9.0.1, 10x Genomics) using the cellranger count pipeline. Reads were aligned to the mouse GRCm39 2024A reference genome provided by 10x Genomics. Seurat (v5.3.0) under R statistical software (version 4.5.0; R Core Team, 2025) was used for quality control, dimensionality reduction, and clustering. For each dataset, we filtered cells based on the following criteria: nFeatureRNA < 200 or > 5,000 and >5% mitochondrial gene content. Raw counts were normalized, scaled, and integrated across samples. The integrated expression matrix was used for all downstream analyses. Principal Component Analysis (PCA) was performed, and the top 20 principal components were used to cluster cells at a resolution of 1.0. Cell clusters were visualized using Uniform Manifold Approximation and Projection (UMAP). Cell type identities were assigned using Seurat’s FindAllMarkers function and by comparing the expression of canonical marker genes. Neutrophils were identified based on canonical markers and subset from the integrated matrix. The neutrophil subset was re-scaled and clustered using the top 10 principal components with a resolution of 0.6. Seurat’s FindAllMarkers function was used to identify marker genes for each neutrophil cluster, and FindMarkers was used to determine differentially expressed genes (DEGs). DEGs were defined using a threshold of log2 fold-change (FC) > 0.5 and adjusted p-value < 0.05. Benjamini-Hochberg method was used to determine the false discovery rate (FDR). To calculate gene signature scores, we used AUCell^4^. Gene sets used for scoring are listed in Supplemental Table 7.

*Isolation of Immune Cells from the Heart and Blood for Flow Cytometry:*

Leukocytes were isolated from the left ventricle (LV) following MI/R. The heart was extracted and perfused with ice-cold phosphate-buffered saline (PBS) to exclude circulating blood cells. The atria and free right ventricular wall were removed and the LV was dissected and minced with fine scissors. The tissue was enzymatically digested in media containing 450U/ml collagenase I (from *Clostridium histolyticum;* Sigma C0130), 60 U/ml Deoxyribonuclease I (DNase I) (from bovine pancreas; Sigma D4513), and 60 U/ml hyaluronidase (from bovine testes; Sigma H3506) dissolved in Dulbecco’s Modified Eagle’s Medium (DMEM) (Sigma D5671) for 60 minutes at 37°C under gentle agitation. Following digestion, cellular digest was kept at 4°C for the remainder of processing. The digestion was passed through a 40 µm cell strainer to exclude cardiomyocytes and debris and centrifuged at 400 x g for 5 minutes. The cell pellet was resuspended in red blood cell (RBC) lysis buffer (Sigma R7757) and incubated at room temperature for 5 minutes. Lysis was stopped by adding cold Hank’s balanced salt solution (HBSS) and cells were centrifuged at 400 x g for 5 minutes. Blood was obtained via the inferior vena cava and transferred into EDTA tubes. Blood leukocytes were isolated by adding prewarmed RBC lysis buffer to blood and incubating at room temperature for 10 minutes. Lysis was stopped by adding cold HBSS and cells were pelleted at 400x g for 5 minutes. Cells isolated from the left ventricle were counted and seeded at a density of 3 x 10^5^ cells. Cells were resuspended in FACS (PBS, 0.02% Fetal Bovine Serum (FBS), and 1mM EDTA) buffer for flow cytometric analysis.

*Flow Cytometric Staining of Immune Cells:*

Isolated cells were resuspended in FACS buffer at a density of 3 x 10^5^ cells. Cells were resuspended in Fc blocking buffer (BD 553142). After blocking, cells were spun down and resuspended in the primary antibody staining buffer, including viability dye to gate out dead cells (eBioscience 65-0866-18). Primary antibodies used for staining are listed in Supplemental Table 9. Cells were fixed and permeabilized using either BD Cytofix/Cytoperm™ Fixation/Permeabilization Solution Kit (BD Biosciences 554714) or Biolegend True-Nuclear™ Transcription Factor Buffer Set (Biolegend 424401) for cytoplasmic or transcription factor staining. Intracellular staining was performed after permeabilization in permeabilization buffer. Annexin V (Invitrogen 88-8005-74) staining was performed according to manufacturer’s protocol. To measure reactive oxygen species (ROS) production, cell suspensions were labeled with Ly6G to mark surface receptors and then incubated with Dihydrorhodamine-123 (DHR123) (Thermo Scientific D23806) at a final concentration of 1:200 for 30 minutes at 37°C. To measure phagocytosis, cells were incubated with Ly6G to label surface receptors and then incubated with 10µg/ml of fluorescent *E. coli* bioparticles (Thermo Scientific P35361). Cells were washed and resuspended for flow cytometry analysis. Cells were acquired on a Cytek Aurora® and analyzed using FlowJo 10. Geometric mean fluorescence intensities (MFIs) were used in analyses. Cells isolated from the left ventricle are expressed as cell number per 3 x 10^5^ cells. Cells isolated from blood are expressed as cells/µl.

*Cyclic Immunofluorescence Staining of STING Activation:*

Hearts were perfused with ice cold PBS to remove excess blood, embedded in TissueTek OCT media, and snap frozen in liquid nitrogen cooled 2-Methylbutane. Hearts were sectioned at 10µm on a Leica Cryostat (CM1950). Slides were equilibrated to room temperature and fixed with 4% PFA. Slides were incubated with 0.3% glycine for 10 minutes, washed with PBS, and blocked with serum-free protein blocking solution overnight. Tissues were subsequently incubated with primary antibodies (MPO Abcam ab300650 (1:50); pSTING Ser366 Invitrogen PA5-105674 (1:200); pTBK1 Ser172 Cell Signaling 54382SF (1:100); diluted in PBS and incubated overnight at 4°C. Following incubation with primary antibody, tissues were washed with PBS and incubated with secondary antibodies (anti-Rabbit Alexa Fluor 488 ab150077, anti-Rabbit Alexa Fluor 647 ab150083, anti-Rat Alexa Fluor 647 ab48272); diluted in PBS for 1 hour at room temperature. Tissues were then washed and incubated with DAPI in PBS for ten minutes. Tissues were washed in PBS and coated with 10% glycerol and sealed with cover slips. Slides were imaged with a Keyence BZ-X810. The images were acquired using the tile scan technique, a method designed to capture high-resolution images of large samples by dividing the area into smaller, overlapping tiles. To improve image quality, background noise was minimized through a two-step correction process. First, images were normalized, and then values from an empty channel were subtracted to eliminate nonspecific fluorescence. After these preprocessing steps, the corrected images from all channels were aligned and merged into a single multichannel image stack, representing the complete dataset for the imaging cycle. Single-cell segmentation was performed using the deep learning-based algorithm Cellpose (version 3.0) with GPU acceleration (17, 18). The “nuclei” model was applied to the DAPI channel from the first imaging cycle (cycle 1) to generate nuclear masks. The expected cell diameter was set to 25 µm (∼66 pixels based on a resolution of 0.37744 µm/pixel), with a flow threshold of 0.8 and a cell probability threshold of 0. To capture cytoplasmic signals, nuclear masks were uniformly expanded by a 25-pixel radius using morphological dilation. Following segmentation, a pixel-level processing pipeline was employed to generate single-cell expression profiles. Using custom Python scripts, the generated cytoplasmic masks were overlaid on the registered, stitched TIFF images from all imaging cycles. For each cell, the mean intensity of each marker (MPO, pSTING, pTBK1) was calculated by aggregating pixel intensities within the cell boundaries. In injured samples, the infarct region was identified using a binary mask derived from the pSTING stain, and only cells within this injury area were retained for downstream analysis.  This process resulted in a comprehensive single-cell dataframe containing expression data and spatial locations for all samples.

*Immunofluorescence Staining of Heart Sections:*

Hearts were perfused with ice cold PBS to remove excess blood, embedded in TissueTek OCT media, and snap frozen in liquid nitrogen cooled 2-Methylbutane. Hearts were sectioned at 10µm on a Leica Cryostat (CM1950). Slides were equilibrated to room temperature and fixed with 4% PFA. For TUNEL and MPO staining, TUNEL staining was performed according to the manufacturer’s protocol (Abcam ab66110). After TUNEL staining was completed, slides were washed and blocked with 10% goat serum and 2% IgG-free BSA in Universal Buffer (0.05 M Tris HCl, pH 7.6, 0.15 M NaCl, and 0.1 % sodium azide) for 1 hour at room temperature. Primary antibody or isotype staining (MPO Abcam ab300650; Rat Isotype Abcam ab18450) diluted in blocking buffer was performed for 1 hour at 37°C in a humidity chamber. Sections were washed and incubated with secondary antibodies (anti-rat Alexa Fluor 647 Invitrogen A21247) and DAPI (Sigma D9542) for 1 hour at room temperature. Tissue sections were then washed with Universal Buffer and mounted using ProLong Diamond Antifade (Thermo Fisher Scientific P36961). Slides were imaged immediately after staining according to the TUNEL manufacturer’s recommendations. For staining of neutrophil extracellular traps, slides were fixed and blocked as stated. Primary antibody or isotype staining (MPO Abcam ab300650; Rat Isotype Abcam ab18450; citH3 Abcam ab281584; Rabbit Isotype Abcam ab172730) diluted in blocking buffer was performed overnight at 4°C in a humidity chamber. Sections were washed and incubated with secondary antibodies (anti-rat Alexa Fluor 647 Invitrogen A21247; anti-rabbit Alexa Fluor 488 Invitrogen A11034) and DAPI (Sigma D9542) for 1 hour at room temperature. Tissue sections were then washed with Universal Buffer and mounted using ProLong Diamond Antifade (Thermo Fisher Scientific P36961). Five-six images were captured from each slide and were acquired as z-stacks using a Zeiss LSM800 Airyscan laser scanning confocal microscope with an EC Plan-Neofluar 40x/1.3 NA objective. For image quantification, maximum intensity projections were generated from the z-stacks and used to quantify the number of TUNEL+ and MPO+ cells per total MPO+ cells and citH3+ and MPO+ cells per total MPO+ cells. Data were analyzed using ImageJ.

*Measure of Intracellular Calcium Flux:*

Neutrophils were isolated from the bone marrow of 10-12 week old male mice using a 62% (v/v) single step Percoll gradient (GE Healthcare) as previously described^41^. Cells were resuspended at 1x10^7^ cells/mL in Hanks balanced salt solution (HBSS)/ 20mM Hepes (H) and loaded with 3µM Indo-1AM (Tocris Bioscience) and 2.5mM probenecid (Molecular Probes) at room temperature for 40 minutes in the dark. Cells were washed and resuspended in cold HBSS/H at 2x10^7^ cells/mL. Cells were aliquoted into fluorescence-activated cell sorter tubes containing HBSS/H with 0.5mM MgCl_2_ and 1mM CaCl_2_ and warmed to 37°C for 1.5 min. Samples were then run on the flow cytometer (Cytek® Aurora) for 30 seconds to establish a baseline reading. Cells were stimulated with 2µM W-peptide (Tocris Bioscience 1800) and the samples analyzed continuously. Neutrophils were labeled with anti-Ly6G and -CD11b. Data were analyzed using FlowJo 10 software. Calcium levels are presented as the ratio of fluorescence emission at 400nm/475nm (calcium bound/calcium unbound) overtime and the area under the curve (AUC)^5^.

*Ex Vivo Efferocytosis Assay:*

To isolate bone marrow derived macrophages (BMDMs), femurs were flushed with ice cold, sterile PBS and filtered through a 40 µm cell strainer. Cells were then pelleted at 400x g for 5 minutes and the pellet was resuspended in RBC lysis buffer and incubated at room temperature for 10 minutes. The pellet was resuspended in macrophage media (RPMI, 2mM GlutaMAX, 1:100 penicillin/streptomycin, 10% FBS, and 100ng/ml murine M-CSF (Biolegend 576404)) and cells were plated at a density of 4.0 x 10^5^ cells/ml 10 cm non-TC treated culture plate. On day 3 of culture, an additional 5 ml of macrophage media was added to the plate. Cells were harvested on day 7 of culture. To obtain neutrophils from the bone marrow, femurs were flushed with ice cold 0.38% sodium citrate in 1x HBSS and filtered through a 40 µm cell strainer. Cells were then pelleted at 400x g for 5 minutes and resuspended in 0.38% sodium citrate in 1x HBSS. To isolate neutrophils from the cell suspension, density centrifugation with a Percoll gradient was used. A solution of 100% Percoll was made by making a 10:1 dilution of Percoll stock solution (Sigma P1644) with 10x HBSS. Dilutions of 72%, 62%, and 52% were made by dilution the 100% Percoll solution in 0.38% sodium citrate in 1x HBSS. The 72% solution was pipetted into a 15 ml conical, and the 62% solution was layered on top, followed by the 52% layer, and the cell suspension. The gradient was centrifuged at 1545 x g for 30 minutes at room temperature. Neutrophils will settle in between the 62% and 72% layers. The neutrophil layer was washed in 0.38% sodium citrate in 1x HBSS and cells were pelleted at 400 x g for 5 minutes. Neutrophils were resuspended in serum-free RPMI at a density of 2.0 x 10^6^ cells/ml and incubated over night at 37°C in 5% CO_2_ to induce apoptosis. Both neutrophils and BMDMs were labeled using Efferocytosis Assay Kit (Cayman Chemicals 601770) according to the manufacture’s protocol. Neutrophils were then incubated with BMDMs at a 4:1 ratio for 6 hours 37°C in 5% CO_2_. Cells were then collected and analyzed via flow cytometry.

*Statistics:*

Unless otherwise stated, data are presented as the mean ± standard deviation (SD). Comparisons between groups were done using Student's unpaired t-test, while comparisons among >2 groups used one‐way or two-way analysis of variance (ANOVA) with Tukey's post-hoc test for multiple pairwise comparisons. Repeated measures two-way ANOVA with Tukey's post-hoc test was used to evaluate longitudinal changes. The assumption of normality was assessed using the Shapiro-Wilk test. A non-significant result (p > 0.05) indicated that the data did not significantly deviate from a normal distribution. If data were not normally distributed, the Wilcoxon rank-sum test was used for comparison. Statistical analysis was performed using GraphPad Prism 10.0.0 (GraphPad Software, Boston, MA), and a p-value <0.05 was considered statistically significant.

**Table 8: Taqman PCR Probes**

| Gene | Primer |
| --- | --- |
| Gapdh | Mm99999915 |
| Ifna1 | Mm03030145 |
| Ifnb1 | Mm00439552 |
| Tnf | Mm00443258 |
| Il6 | Mm00446190 |
|  |  |

| Marker | Clone | Fluorophore | Catalog Number | Dilution |
| --- | --- | --- | --- | --- |
| CCR2 | SA203G11 | Brilliant Violet 421 | Biolegend 150605 | 1:100 |
| CD11b | GL-1 | PerCP | Biolegend 101229 | 1:100 |
| CD45 | 30-F11 | Alexa Fluor 700 | Biolegend 103127 | 1:200 |
| CD64 | X54-5/7.1 | Brilliant Violet 711 | Biolegend 139311 | 1:50 |
| CD206 | C068C2 | PE-Fire 700 | Biolegend 141741 | 1:100 |
| CXCR2 | SA044G4 | APC | Biolegend 149312 | 1:50 |
| CXCR4 | L276F12 | PE-Dazzle 594 | Biolegend 146514 | 1:100 |
| Ly6G | 1A8 | APC-Fire 750 | Biolegend 127651 | 1:50 |
| pIRF3 | D6O1M | APC | Cell Signaling 81093 | 1:50 |
| F4/80 | BM8 | Brilliant Violet 711 | Biolegend 123147 | 1:100 |
| CD47 | miap301 | PE-Cy7 | Biolegend 127524 | 1:100 |
| MertK | 2B10C42 | PE | Biolegend 151506 | 1:50 |
| CD18 | M18/2 | PE | Biolegend 101407 | 1:100 |
| CD63 | NVG-2 | APC | Biolegend 143905 | 1:100 |

**Table 9: Flow Cytometry Antibodies**

**Table 10: Characteristics of Bulk-RNA Sequencing Donors**

| Sex (n, %) | |
| --- | --- |
| Male | n=20; 50% |
| Female | n=20; 50% |
| Race (n,%) | |
| Caucasian | n=28; 70% |
| African American | n=3; 7% |
| Asian | n=4; 10% |
| Hispanic/Latino | n=5; 13% |

| Sample | Age | Sex | Biopsy Location | Disease |
| --- | --- | --- | --- | --- |
| P1 | 44 | Male | LV free wall | Control |
| P7 | 55 | Female | LV free wall | Control |
| P3 | 57 | Male | LV free wall | Acute MI of the anterior LV |
| P9 | 52 | Female | LV free wall | Subacute inferolateral MI |

**Table 11: Characteristics of Infarct Patient Samples**

**Supplemental Figure 1.** **Genotype and baseline phenotype of neutrophil-specific STING KO mice: (A)** Neutrophil-specific knockout mice were bred by crossing mice heterozygous for Cre recombinase under the Ly6G promoter (Hasenberg, *Nature Methods*, 2015) with mice homozygous for floxed STING purchased from Jackson Labs (#031670). **(B)** The wildtype Ly6G allele appears at 324bp; the Ly6G knock-in allele appears at 373bp; the wildtype STING allele appears at 298bp and the STING floxed allele appears at 450bp **(C)** Complete blood count (CBC) analysis of wild-type and neutrophil-specific STING KO mice show no significant differences in cell counts. **(D)** Weights of male and female mice show no significant difference between genotypes**.** Data are presented as mean ± SD **(C-D).** Statistical analyses were conducted using an unpaired t test **(C-D).**

**Supplemental Figure 2. Purity assessment of neutrophils isolated by density gradient to confirm STING knockout:** Flow cytometry analysis of the neutrophil (top) and non-neutrophil (bottom) fractions obtained by density gradient centrifugation was performed to confirm purity during assessment of STING knockout efficiency and specificity. Cells are gated on single and live cells (not shown). The neutrophil fraction was ~97% pure, with minimal contamination from CD64+ cells (~2.5%) and less than 1% CD115+ or CD11b- cells. The non-neutrophil fraction contained ~62.6% CD11b+, Ly6G- cells, with ~26% CD64+ cells, ~13% CD115+ cells, and ~6.5% CD11b- cells.

**Supplemental Figure 3.** **Cardiac injury is unaffected by loss of STING in neutrophils in female mice: (A)** Representative echocardiography images of parasternal long-axis left ventricular M-mode in female WT and neuSTING KO mice. **(B-C)** Female neuSTING KO mice show no difference in LVESD and LVEDD or ejection fraction and fractional shortening compared to WT mice. (n=5 per group). **(D)** Ratios of ventricular weight to tibia length show no difference in cardiac hypertrophy in female neuSTING KO mice compared to WT mice. (n=5 per group). **(E-F)** Masson’s Trichrome staining no difference in LV fibrosis in female neuSTING KO mice compared to WT mice. (n=5 per group). **(G)** Quantification of serum NT-proBNP 14 days after reperfusion; (n=5 per group). Scale bars equal 2.5 mm. Data are presented as mean ± SD **(B-E)**. Statistical analyses were conducted using an unpaired t test **(D-E)** two-way repeated measures ANOVA with Tukey's post-hoc test **(B-C).** ^#^p<0.05; ^##^p<0.01; ^###^p<0.001; ^####^p<0.0001. vs WT baseline, ^$^p<0.05; ^$$^p<0.01; ^$$$^p<0.001; ^$$$$^p<0.0001. vs KO baseline. LV = left ventricle; LVESD = left ventricular end systolic diameter; LVEDD = left ventricular end diastolic diameter; EF = ejection fraction; FS = fractional shortening; VW/TL = ventricular weight/ tibia length.

**Supplemental Figure 4. Analysis of neutrophils in wildtype male and female mice after MI/R: (A)** Flow cytometry analysis of total neutrophils (CD45^+^, Ly6G^+^) isolated from cardiac tissue 24 hours after MI/R compared to sham mice in WT make and female mice; (n=4-5 per group). **(B)** STING activation in neutrophils, as measured by IRF3 phosphorylation at S396, isolated from cardiac tissue 24 hours after MI/R compared to sham mice in WT male and female mice; (n=4-5 per group). Data are presented as mean ± SD. Statistical analysis was conducted using one-way ANOVA with Tukey's post-hoc test. *p<0.05; **p<0.01; ***p<0.001; ****p<0.0001.

**Supplemental Figure 5. Cell type marker genes in single-cell RNA sequencing analysis:** Dot plot showing the marker genes for each cell type identified in single-cell RNA sequencing analysis.

**Supplemental Figure 6. AUCell scores for neutrophil effector functions: (A)** Comparison of calcium signature scores in cardiac neutrophils from WT and neuSTING KO mice 24 hours after MI/R and calcium signature scores projected onto UMAPs of WT and neuSTING KO neutrophils. **(B)** NET signature scores projected onto UMAPs of WT and neuSTING KO neutrophils. **(C)** Comparison of degranulation signature scores in cardiac neutrophils from WT and neuSTING KO mice 24 hours after MI/R and degranulation signature scores projected onto UMAPs of WT and neuSTING KO neutrophils. **(D)** Comparison of phagocytosis signature scores in cardiac neutrophils from WT and neuSTING KO mice 24 hours after MI/R and phagocytosis signature scores projected onto UMAPs of WT and neuSTING KO neutrophils. **(E)** Comparison of ROS signature scores in cardiac neutrophils from WT and neuSTING KO mice 24 hours after MI/R and ROS signature scores projected onto UMAPs of WT and neuSTING KO neutrophils. Data are presented as the median (center line), interquartile range (IQR) (box), and 1.5× IQR (whiskers); individual points represent single cells. Statistical analyses were conducted Wilcoxon rank-sum test. *p<0.05; **p<0.01; ***p<0.001; ****p<0.0001.

**Supplemental Figure 7. Gating strategy for flow cytometry experiments:** Gating strategy used in flow cytometry time course experiments. Cells are gated on single and live cells (not shown). CD11b was plotted against CD45 to identify CD45+, CD11b+ myeloid cells. Subsequently, CD11b was plotted against Ly6G to identify neutrophils. CD45+, CD11b+, Ly6G+ (neutrophils) cells were then used to gate CXCR4+ or CXCR2+ neutrophils. CD45+, CD11b+ cells were also used to plot CD64 against Ly6G to identify macrophage and monocyte populations as CD45+, CD11b+, Ly6G-, CD64+. Monocytes and macrophages were further gated on CCR2+ , CD206+, and MertK+.

**Supplemental Figure 8. *In vivo* and *ex vivo* efferocytosis assays: (A)** Expression of CD47 on peripheral blood neutrophils from WT or neuSTING KO mice at baseline; (n=3 per group) **(B)** Gating of F4/80 and Ly6G shows a Ly6G+, F4/80- population and a F4/80+, Ly6G+ indicating a distinct Ly6G+ population and a Ly6G+ populatioin associated with F4/80+ macrophages. **(C)** Apoptosis signature scores projected onto UMAPs of WT and neuSTING KO neutrophils. **(D)** Schematic of experimental design for *ex vivo* efferocytosis assay. Neutrophils were isolated from the bone marrow of WT or neuSTING KO mice, labeled with CFSE, and incubated overnight in serum-free media to induce apoptosis. Subsequently, apoptotic neutrophils were collected and incubated with WT bone marrow-derived macrophages (BMDMs) for six hours. Efferocytosis was then assessed by flow cytometry; Annexin V staining of WT and neuSTING KO neutrophils shows neuSTING KO neutrophils were less apoptotic compared to WT neutrophils. (n=3 per group); Assessment of efferocytosis by flow cytometry shows neuSTING KO neutrophils are efferocytosed less than WT neutrophils. (n=3 per group). Data are presented as mean ± SD. Statistical analyses were conducted using an unpaired t test. *p<0.05; **p<0.01; ***p<0.001; ****p<0.0001.

**Supplemental Figure 9. Single cell RNA sequencing of neuSTING KO macrophages reveals increased stressed phenotype after MI/R: (A)** UMAP plots of cardiac macrophages from WT and neuSTING KO mice 24 hours after MI/R. **(B)** Proportions of macrophage subclusters in cardiac macrophages from WT and neuSTING KO mice. **(C)** Heatmap of top marker genes across each macrophage subcluster. **(D)** Gene set enrichment analysis for GO Biological Processes of top marker genes in macrophage subclusters. FDR adjusted p-value and the size of the dot corresponds to the counts of genes in the indicated pathway. **(E)** Volcano plot of differentially expressed genes in cardiac macrophages from neuSTING KO mice relative to WT mice. P values were corrected for multiple tests using the FDR method; criteria for differential expression were log2FC >0.5 and adjusted p value <0.05. Significantly upregulated genes are plotted in red and significantly downregulated genes are plotted in blue.

**References:**

1.	Hasenberg A, Hasenberg M, Männ L, Neumann F, Borkenstein L, Stecher M, Kraus A, Engel DR, Klingberg A, Seddigh P, Abdullah Z, Klebow S, Engelmann S, Reinhold A, Brandau S, Seeling M, Waisman A, Schraven B, Göthert JR, Nimmerjahn F and Gunzer M. Catchup: a mouse model for imaging-based tracking and modulation of neutrophil granulocytes. *Nature Methods*. 2015;12:445-452.

2.	Ackers-Johnson M, Li PY, Holmes AP, O'Brien SM, Pavlovic D and Foo RS. A Simplified, Langendorff-Free Method for Concomitant Isolation of Viable Cardiac Myocytes and Nonmyocytes From the Adult Mouse Heart. *Circ Res*. 2016;119:909-20.

3.	Tejada T, Tan L, Torres RA, Calvert JW, Lambert JP, Zaidi M, Husain M, Berce MD, Naib H, Pejler G, Abrink M, Graham RM, Lefer DJ, Naqvi N and Husain A. IGF-1 degradation by mouse mast cell protease 4 promotes cell death and adverse cardiac remodeling days after a myocardial infarction. *Proc Natl Acad Sci U S A*. 2016;113:6949-54.

4.	Aibar S, González-Blas CB, Moerman T, Huynh-Thu VA, Imrichova H, Hulselmans G, Rambow F, Marine J-C, Geurts P, Aerts J, van den Oord J, Atak ZK, Wouters J and Aerts S. SCENIC: single-cell regulatory network inference and clustering. *Nature Methods*. 2017;14:1083-1086.

5.	Kinsella RL, Sur Chowdhury C, Smirnov A, Mreyoud Y, Kimmey JM, Esaulova E, McKee SR, Pride A, Kreamalmeyer D, Artyomov MN and Stallings CL. ATG5 suppresses type I IFN-dependent neutrophil effector functions during Mycobacterium tuberculosis infection in mice. *Nature microbiology*. 2025;10:1323-1339.
